# Psychological distress and productivity loss: a longitudinal analysis of Australian working adults

**DOI:** 10.1007/s10198-025-01764-9

**Published:** 2025-04-30

**Authors:** Syed Afroz Keramat, Tracy Comans, Alison Pearce, Rabeya Basri, Rubayyat Hashmi, Nadeeka N. Dissanayaka

**Affiliations:** 1 Centre for Health Services Research, Faculty of Health, Medicine and Behavioural Sciences, The University of Queensland, Brisbane, QLD 4102 Australia; 2 The Daffodil Centre, and Sydney School of Public Health, The University of Sydney, Sydney, NSW Australia; 3 Department of Economics, University of Rajshahi, Rajshahi, Bangladesh; 4 The Australian Centre for Housing Research, The University of Adelaide, Adelaide, South Australia 5000 Australia; 5 The University of Queensland Centre for Clinical Research (UQCCR), Faculty of Health, Medicine and Behavioural Sciences, The University of Queensland, Brisbane, Australia; 6 The ALIVE National Centre for Mental Health Research Translation, The University of Melbourne, Melbourne, VIC 3010 Australia

**Keywords:** Psychological distress, Productivity loss, Absenteeism, Presenteeism, Underemployment, Australia

## Abstract

By 2030, it is anticipated that poor mental health will cost the global economy approximately $6 trillion per year, primarily due to productivity loss. It is crucial to understand how psychological distress contributes to productivity loss in the workplace. We aim to investigate the relationship between psychological distress and productivity loss in the Australian working population. We utilized eight waves of longitudinal data drawn from the Household, Income, and Labour Dynamics in Australia (HILDA) Survey (waves 7, 9, 11, 13, 15, 17, 19, and 21). We compiled an unbalanced panel data set comprising 70,973 person-year observations from 18,729 unique working adults. We used Fixed-effects Poisson regression and Fixed-effects logistic regression models to investigate the within-person differences in the relationship between psychological distress and productivity loss (measured through sickness absence, presenteeism, and underemployment). We found that moderate and high psychological distress is associated with a higher rate of sickness absence, presenteeism, and underemployment when a working adult shifted from low psychological distress after controlling socio-demographic, health, and employment-related characteristics. Our study demonstrated that moderate to high psychological distress adversely affected employees’ job productivity through increased sickness absence, a higher likelihood of presenteeism, and greater levels of underemployment. Our findings also revealed that employees with moderate and high psychological distress incurred additional annual sickness absence costs of AUD 60.66 and AUD 99.26, respectively, compared to peers with low psychological distress. Additionally, our study found that employees with moderate and high levels of psychological distress experienced significantly higher levels of presenteeism, which resulted in additional annual costs of AUD 1,166.30 and AUD 3,656.05, respectively, compared to their counterparts with low psychological distress. Psychological distress imposed significant costs on Australian workplaces. Implementing workplace health promotion programs should be prioritized as a policy to address psychological distress among employees, enhance their well-being, and improve overall productivity.

**Supplementary Information:**

The online version contains supplementary material available at 10.1007/s10198-025-01764-9.

## Introduction

Mental health disorders such as anxiety and depression [1], which contribute significantly to the global burden of diseases [2]. Psychological distress refers to unpleasant emotions, often characterized by symptoms related to depression and anxiety [1, 3]. It can be used to assess an individual’s state of mental health and well-being [4]. The costs of mental health disorders around the world each year is estimated to be $2.5 trillion, with depression and anxiety making up about $1 trillion of that total [5]. With the persistent increase in mental health disorders across the world [2], its associated economic burden is expected to reach up to $6 trillion annually by 2030 [5]. The greatest part of the costs associated with mental health disorders is indirect costs (e.g., productivity loss) [6, 7]. In the US, for instance, the economic burden of adults with major depressive disorder in 2018 was estimated at $326.2 billion, of which indirect costs associated with work and productivity (absenteeism and presenteeism) accounted for 61% (198.6 billion dollars) of the total costs [7].

In 2020–2021, an estimated 15.4% of Australians aged 16–85 years experienced high or very high levels of psychological distress [8], which was relatively higher than the 13% and 12% recorded in 2017–2018 and 2014–2015, respectively [9]. Young adults (16–34 years) comprise the majority of the Australian civil workforce and had the highest rate of psychological distress (20%) in 2020–2021 [8]. As a result, there is an urgent need for interventions to address the growing level of psychological distress among the Australian population and limit its effects on productivity. Recent estimates suggest that improving the quality of life of people with mental health problems in Australia could increase economic participation and contribute $1.3 billion to the Australian economy each year [10].

Productivity loss attributable to workers’ physical and psychological ill health continue to gain the attention of researchers, policymakers, and industries worldwide [11]. Sickness absence, which refers to an employee’s absence from work due to ill health, is often used as the main measure of productivity loss [12]. The absence of employees from work reduces organizational output and disrupts organizational functioning [13], especially when the absent worker is not replaced at all or is replaced with a less efficient worker [14]. Absenteeism also creates a physical and psychological burden on other workers who may have to take on the additional responsibility of the absent worker [15], perhaps increasing the risk of absenteeism among other workers. Evidence suggests that workers with increased levels of psychological distress often have a high absenteeism rate, thereby contributing to productivity loss [15, 16]. Although sickness absence is commonly used as the main measure of productivity loss, researchers have a growing consensus that presenteeism contributes to an even greater loss of productivity [15, 17]. Presenteeism refers to showing up for work despite being physically or psychologically ill [12]. Presenteeism is increasingly gaining global recognition due to its high prevalence and associated loss of productivity [12, 17]. Psychological distress has been identified as one of the strongest correlates of presenteeism among workers [18]. The reduced productivity due to presenteeism is often associated with manifestations of anxiety and depression [19] which are the key features of psychological distress. Underemployment is a multidimensional concept that often involves part-time work, work hour constraints, underutilization of skills, and working at a lower level than actual qualification [20, 21]. Underemployment is often used as a measure of spare capacity in the labour market. Greater levels of underemployment indicate that workers are unable to work more and generate sufficient income to meet their needs. Despite the increasing trends of underemployment [22], there are limited explicit studies examining the relationship between psychological distress and underemployment.

Productivity is a key area that is affected by chronic conditions. The most common measure of productivity includes sickness absence, presenteeism, underemployment, labour force status, and ability to participate in work. Existing evidence suggests that mental health-related chronic conditions have a substantial effect on reduced work productivity. Further study is required to measure the effects of a specific mental health condition (e.g., psychological distress) on productivity loss. Therefore, we aim to examine the association between psychological distress and productivity loss measured through sickness absence, presenteeism, and underemployment. We used nationally representative longitudinal data to examine the within-person differences in the association between psychological distress and productive loss in an Australian working population.

## Methods

### Data source

Annual data from The Household, Income, and Labour Dynamics in Australia (HILDA) Survey were used to conduct this empirical analysis. The HILDA Survey is a nationally representative household panel study that began in 2001 and has been conducted annually thereafter [23]. The Melbourne Institute: Applied Economic and Social Research at the University of Melbourne oversees designing, administering, and preparing data for release, and the Australian Government sponsors it through the Department of Social Services [23]. The HILDA Survey used multistage clustered stratified sampling technique to collect data annually from over 13,000 individuals residing in more than 7,000 households. The survey collects information from the Australian adult population on a wide range of topics. Rotating modular content includes questions on demographics, socio-economic, fertility, family life, health, and labour market activity. Data were collected from eligible adult household members (aged 15 years and over) using a combination of self-completion questionnaires and in-person or over-the-telephone interviews conducted by experienced interviewers. A detailed description of the survey has been provided elsewhere [23]. The HILDA Survey data is ideal for conducting the present study as it comprises detailed information on key outcome variables (sickness absence, presenteeism, and underemployment), primary variable of interests (psychological distress), and other covariates (socio-demographic, health-related, and job-related characteristics) at multiple time points.

### Analytic sample and missing data

**Fig. 1:**
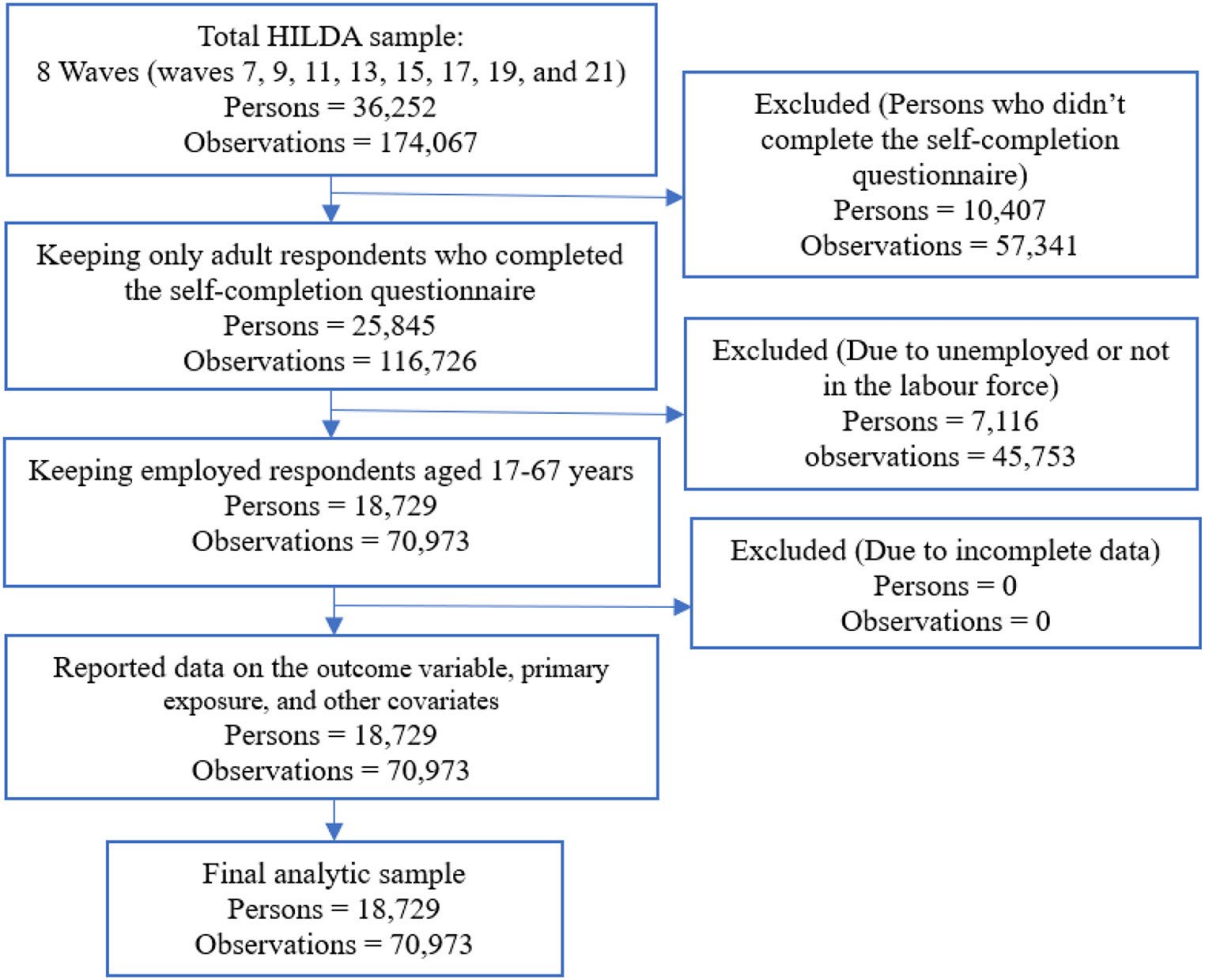
Participants flow into the analytic sample and missing data

Data on our main variable of interest (psychological distress) were collected in a rotating module, so was only available in a limited number of waves. Therefore, we utilized the most recent eight waves of data (waves 7, 9, 11, 13, 15, 17, 19, and 21) spanning the period between 2007 and 2021. One of the major survey instruments through which all adult participants in the selected household were interviewed in the HILDA Survey is the self-completion questionnaire (SCQ). In the SCQ, respondents were asked to report health and well-being (e.g., by using SF-36 health survey, and Kessler Psychological Distress Scale [K10]), health behaviours (smoking, drinking, exercise, weight, diet), social capital/relationships (satisfaction with family, social support, community participation, religion), neighbourhood characteristics, life events, finances (stressful events, savings habits, risk preference, household expenditure), job attributes, parenting (parenting stress/work family gains and strains), attitudes to work / gender roles/marriage, and personality. If a respondent did not complete the SCQ, we excluded them from the final analytic sample. Given our interest is the effects of psychological distress on productivity loss, we restricted our study sample to individuals who were currently employed. To ensure a focused analysis of the working-age population, we restricted our sample to individuals aged 17 to 67 years, encompassing the typical working age range in Australia. This age range (17–67 years) was chosen to include individuals at both ends of the working age spectrum, from young adults just entering the labour market following education to older adults approaching retirement. After applying the inclusion criteria, our final analytic sample encompassed 70,973 annual observations from 18,729 unique respondents. We did not exclude participants due to missing data on the studied variables (e.g., psychological distress, sickness absence, presenteeism, and underemployment) across all eight waves. This ensures the sample from the nationally representative survey is representative. We handled missing information in two ways. Firstly, we imputed the missing information of a respondent by their previous or next wave information. To address the remaining missing data, we employed simple imputation techniques, using the mean for continuous variables and the mode for categorical variables. The flow of the respondents into the analytic sample and missing data is presented in Fig. 1.

### Measures

#### Outcome variables

The key outcome variables of our study are sickness absence (count variable), presenteeism (binary variable), and underemployment (binary variable). Sickness absence is a self-reported measure of the number of paid sick leave taken in the prior 12 months. The survey asked respondents, ‘During the last 12 months, have you taken any paid sick leave?’ and if so, ‘How many weeks or days did you spend on paid sick leave?’. The responses were discrete as they consisted of non-negative integers, including zero.

The variable presenteeism was constructed from the Short Form (SF-36) Health Survey as part of the SCQ in the HILDA Survey. Individuals were asked whether they have experienced any of the following in the previous four weeks as a result of emotional issues or due to any physical problems: “cutting down on the amount of time you spent on work or other activities,” “accomplished less than you would like,” and “didn’t perform work or other activities as carefully as usual”. The responses to the above questions were recorded in binary form. We constructed a binary indicator for presenteeism with the value of one for “Yes” answers to any of the above three questions, and zero otherwise.

To assess presenteeism, we also employed an alternative measure. As an alternative to the existing measure of presenteeism, we utilized self-reported data on the number of days worked despite feeling unwell to capture the effects of psychological distress on productivity at work. To measure presenteeism, wave 21 of the HILDA Survey included a question asking respondents, “How many days during the past four weeks did you work despite feeling either physically or mentally unwell?” The data on the number of days worked while unwell were discrete, taking on non-negative integer values, including zero.

We developed our measure of underemployment on the definition of the Australian Bureau of Statistics (ABS) definition, where the actual number of hours worked per week is less than the hours would like to work per week. The HILDA Survey asks all working adults how many hours each week they normally work in all occupations and would prefer to work, considering the impact on their income. We constructed a binary variable from this, defining a person as underemployed if they reported a desire to work more hours and were currently employed and working fewer than 35 h per week on average (the regular full-time work week, as defined by the ABS).

#### Exposure variable

The main variable of interest of our study is psychological distress. The Kessler 10 psychological distress Scale (K10) is a 10-item unidimensional scale developed to assess psychological distress [24]. The K10 questionnaire asks respondents how frequently they experienced certain emotions (e.g., tired out of no good reason, nervousness, hopelessness, restlessness, anxiety, melancholy, and worthlessness) over the past 30 days. Each item is scaled from 1 (none of the time) to 5 (all of the time), and the total score is used as an indicator of mental distress. The resulting score ranges from 10 to 50. High scores imply severe psychological distress, whereas low scores indicate low distress. The survey categorized psychological distress into four groups using the K10 scores: low (10–15), moderate (16–21), high (22–29), and very high (30–50). We re-classified psychological distress as low, moderate, and high (by merging high and very high). We measured the probability of transitioning from one group to another group of psychological distress over time. The key reason for reporting the transition probability is to provide insight into whether the key exposure variable has sufficient variability to support the Fixed-effects analysis.

#### Other covariates

We incorporated several potential confounding variables that are significant correlates of workplace productivity based on prior research [25, 26]. Age (17–24, 25–54, and 55–67 years), relationship status (unpartnered and partnered), highest education level completed (year 12 and below, professional qualifications, university degrees), household yearly disposable income (quintile 1 [poorest] to quintile 5 [richest]), and region of residence (major city, regional city, and remote area) are the socio-demographic factors considered in our study. Additionally, we included two measures of health-related characteristics (weight category measured through Body Mass Index (BMI) and disability), and three health-related behavioural characteristics: smoking status (non-smoker and current smoker), alcohol consumption (non-drinker and current drinker), and physical activity (less than the recommended level and recommended level). Furthermore, we incorporated a variety of work-related factors as potential confounders, including firm size, employment contract, occupation, supervisory responsibilities, union membership, paid holiday leave, sick leave, and overall job satisfaction. More details regarding the control variables can be found in the Appendix (Table A1).

### Estimation strategy

While psychological distress can arise from various causes, we use theoretical frameworks to guide our understanding of how workplace factors may contribute to psychological distress and productivity loss.

#### Theoretical framework

Several theoretical models have been used to aid our understanding of sickness absence, presenteeism, and underemployment in relation to productivity loss. Commonly used models for absenteeism and presenteeism include the job demands-control model [27], and the effort-reward imbalance at work model [28]. Meanwhile, the relative deprivation theory [29] is often used to explain the phenomenon of underemployment and productivity. The job demands-control model posits that high job strain often occurs in organizations where there are increased job demands but low job control [27]. Therefore, despite having difficult tasks to accomplish, the worker has no decision-making latitude in carrying out tasks. Evidence suggests that while organizational managers are often interested in maximizing productivity by increasing workload, such endeavors are often associated with worker exhaustion and poor work attitude if the increased workload does not bring commensurate high job control or decision-making capacity [30]. As a result, the imbalance between job demands and job controls tends to affect employees’ physical and psychological health, thereby increasing the propensity for absenteeism and presenteeism, and consequently loss of productivity [30].

Workers’ output is often influenced by intrinsic factors such as rewards and self-esteem and extrinsic factors including workload and job requirements [31, 32]. According to the effort-reward imbalance model, work-related stress persists among workers when high effort at work is often less rewarded [28]. Therefore, the perception that workers’ output would not be rewarded adequately often results in psychological distress and contributes to productivity loss through absenteeism and presenteeism [33]. The relative deprivation theory has often been used to explain psychological distress associated with underemployed [29]. Relative deprivation refers to a perceived discrepancy between people’s work expectations and their actual status at work. The theory contends that the lack of congruence between employees’ expectation for a job and their actual engagement at work often results in negative emotional reactions [34] which contribute to negative job attitudes and psychological distress [35]. For instance, employees who work in organizations where they do not fully utilize their skills have persistently lower job attitudes and poorer psychological well-being [35, 36], which could contribute to the loss of productivity.

#### Model estimation: sickness absence

We employed Fixed-effects Poisson regression model to analyse sickness absence, a non-negative integer or count variable, measured as the number of paid sick leave taken in the past 12 months. This approach helps minimize the probability of omitted variable bias resulting from unobserved factors that have an impact on the outcome [37]. To estimate the parameters of the model, we utilize the conditional maximum likelihood method proposed by [38]. This approach allows us to assess the relationships between the explanatory variables and sickness absence while controlling for individual-specific heterogeneity.

A random variable $\:{Y}_{i}$ is considered to follow a Poisson distribution with parameter $\:{\lambda\:}_{i}$, which is related to the regressors $\:{x}_{i}$. The Poisson distribution function is:

1 $$Pr\>\left[ {Y = {y_i}\left| {{x_i}} \right.} \right] = {{{e^{ - \lambda {\>_i}}}\>\lambda \>_i^{{y_i}}} \over {{y_i}!}},\,\,\,\,\,\,\,\,\,\>{y_i} = 0,\,1,\,2, \ldots \ldots $$

Where, $\:\lambda\:$ is the intensity or rate parameter. The mean and variance of this distribution can be demonstrated to be$\:\:E\:\left[Y\right]=Var\:\left[Y\right]=\lambda\:$.

The Poisson regression model can be estimated using maximum likelihood techniques. The log-likelihood Poisson function is:

2 $$\:{ln}L={\sum\:}_{i=1}^{n}\:\left\{-{\lambda\:}_{i}+{y}_{i}{x}_{i}^{/}\beta\:-{ln}{y}_{i}!\right\}$$

Considering a linear case, the individual-specific effect is incorporated additively. Therefore, $\:{\mu\:}_{it}={\alpha\:}_{i}+{x}_{it}^{/}\beta\:$. However, the individual-specific effect is introduced multiplicatively when dealing with count data. In models with an exponential conditional mean,$\:{\:exp\:}({x}_{it}^{/}\beta\:)$, specifying a model with a multiplicative individual-specific effect $\:{\alpha\:}_{i}$, denotes the conditional mean to be:

3 $$E\left[ {{y_{it}}|{x_{it}},\alpha } \right] = {\mu _{it}} = {\alpha _{it}}{\lambda _{it}} = {\alpha _i}\exp (x_{it}^/\beta )$$

Where, $\:\alpha\:$ represents the individual-specific effect. In the Fixed-effects model, there is a possibility of correlation between the individual-specific effects $\:{\alpha\:}_{i}$ and the regressors$\:{\:x}_{it}$.

The Fixed-effects Poisson model is the easiest parametric approach for analysing count data, where, conditional on $\:{\lambda\:}_{it}$ and individual-specific effects$\:{\:\alpha\:}_{i}$,

4 $$\:{y}_{it}\approx\:P\:\left[{\mu\:}_{it}={\alpha\:}_{i}{\lambda\:}_{it}\right]$$

Where, $\:{\lambda\:}_{it}\:$is a specified function of $\:{x}_{it}$ and$\:\:\beta\:$, and in certain cases, we focus on the exponential form presented in Eq. (3) as a specialized approach. When employing maximum likelihood estimation, both the individual-specific effects, $\:{\alpha\:}_{i}$, $\:i=$1,…., $\:n$, and$\:\:\beta\:$ are treated as parameters that are estimated together.

The estimation of Fixed-effects Poisson regression involves maximizing the conditional log-likelihood. This estimation method, known as conditional maximum log-likelihood estimation, was originally derived to estimate the Fixed-effects Poisson estimator [39]. The conditional maximum log-likelihood function has been explicitly defined in our study as stated below.

5 $$\begin{aligned}{L_c}\left( {\beta \:} \right) & = \sum \: _{i = 1}^n\left[ {ln\:\left( {\sum \: _{t = 1}^T{y_{it}}} \right)! - \sum \: _{t = 1}^Tln\:\left( {{y_{it}}!} \right)} \right. \\ & \quad \left. { + \sum \: _{t = 1}^T{y_{it}}ln\left( {\frac{{exp\:\left( {x_{it}^/\beta \:} \right)}}{{\sum \: _{s = 1}^Texp\:\left( {x_{is}^/\beta \:} \right)}}} \right)} \right] \\ \end{aligned} $$

#### Model estimation: presenteeism and underemployment

To investigate the association between psychological distress and productivity loss, our study employs a Fixed-effects logistic regression model as the outcome variables, presenteeism and underemployment, are both binary variables. Fixed-effects logistic regression is utilized when there is a need to control for unobserved heterogeneity in analysing binary or categorical outcomes. By including fixed-effects, the model accounts for the individual-specific characteristics that remain constant over time and may influence the outcome variable. The use of Fixed-effects logistic regression helps addressing omitted variable bias and controls for the unobserved factors that can confound the relationship between the predictors and the binary outcome variable. Therefore, we hypothesized that the following logistic functional forms might be used to describe presenteeism and underemployment-related productivity loss caused by psychological distress.

6 $$\:{PS}_{it}^{*}={\Gamma\:}\:({PD}_{it},{HF}_{it},{JF}_{it},{D}_{it},\:{\sigma\:}_{i},\:{\epsilon\:}_{it})$$

7 $$\:{UE}_{it}^{*}={\Gamma\:}\:({PD}_{it},{HF}_{it},{JF}_{it},{D}_{it},\:{\sigma\:}_{i},\:{\epsilon\:}_{it})$$

Where $\:{PS}_{it}^{*}$ is the continuous latent response of an individual showing the likelihood of experiencing presenteeism at time t. Likewise, $\:{UE}_{it}^{*}$ is the continuous latent probability that individual $\:i$ would experience underemployment at time $\:t$. The explanatory variables $\:{PD}_{it}$ is an ordinal indicator of the level of psychological distress; $\:{HF}_{it}$ is a vector of health-related factors and behaviours, $\:{JF}_{it}$ is a vector of job-related factors, $\:{D}_{it}$ is a vector of demographic controls; $\:{\sigma\:}_{i}$ is unobserved time-invariant heterogeneity; and $\:{\epsilon\:}_{it}$ is the error term. The subscript $\:it$ refers to individual $\:i$ and time $\:t$, respectively.

Since, $\:{PS}_{it}^{*}$ is unobserved, the observed response is as follows:

8 $$\:{PS}_{it}=\left\{\begin{array}{c}1\:,\:\:if\:{PS}_{it}^{*}>0\\\:0,\:\:otherwise\end{array}\right.$$

Similarly, the observed response for $\:{UE}_{it}^{*}$ is as follows:

9 $$\:{UE}_{it}=\left\{\begin{array}{c}1\:,\:\:if\:{UE}_{it}^{*}>0\\\:0,\:\:otherwise\end{array}\right.$$

We used Hausman tests to examine the correlation between unobserved heterogeneity and the explanatory variables, and the results indicate a significant correlation between them [40]. The findings indicate that employing Fixed-effects logistic regression models is more suitable compared to Random-effects logistic regression models, which assumes that there is no correlation between unobserved heterogeneity and the explanatory variables [41]. Therefore, we employed the Fixed-effects logistic model [42] for modelling presenteeism and underemployment. The model is defined as follows:

10 $$\eqalign{& log\left( {{{Pr\left( {P{S_{it}} = 1} \right)} \over {1 - Pr\left( {P{S_{it}} = 1} \right)}}} \right) = \theta {\>_0} + \theta {\>_1}P{D_{it}} + \theta {\>_2}H{F_{it}} \cr & + \theta {\>_3}J{F_{it}} + \theta {\>_4}{D_{it}} + \theta {\>_5}T{V_{it}} + \theta {\>_6}T{C_i} + \alpha {\>_i} + \xi {\>_{it}} \cr} $$

11 $$\eqalign{& log\left( {{{Pr\left( {U{E_{it}} = 1} \right)} \over {1 - Pr\left( {U{E_{it}} = 1} \right)}}} \right) = \phi {\>_0} + \phi {\>_1}P{D_{it}} + \phi {\>_2}H{F_{it}} \cr & + \phi {\>_3}J{F_{it}} + \phi {\>_4}{D_{it}} + \phi {\>_5}T{V_{it}} + \phi {\>_6}T{C_i} + \lambda {\>_i} + \xi {\>_{it}} \cr} $$

With $$\:{\xi\:}_{i\:\sim}\:N(0,{\sigma\:}_{\alpha\:}^{2})$$; $$\:{\theta\:}_{i}$$ and $$\:{\phi\:}_{i}$$$$\:\left[i=1\dots\:6\right]$$ are vectors of parameters to be assessed. TV is a vector of time-varying variables; TC is a vector of observed time-constant variables; $$\:{\alpha\:}_{i}$$ and $$\:{\lambda\:}_{i}$$ is unobserved heterogeneity which is constant for individuals; and$$\:\:{\xi\:}_{it}$$ is the error term.

As with our analysis of absenteeism, the core parameter of interest $\:{\theta\:}_{1}$ and $\:{\phi\:}_{1}$ represent the impact of productivity loss owing to psychological distress (variable$\:{\:PD}_{it}$). We anticipate a greater likelihood of productivity loss with a higher level of psychological distress.

#### Robustness check

Conducting a robustness check using an alternative regression technique is a common practice to verify the validity and stability of the main study findings. This involves re-estimating the main regression model using a different method to check if the results hold. To ensure the robustness of our findings, we conducted a sensitivity analysis using an alternative regression technique. We have fitted a Random-effects regression model to verify the stability of results and identify the between-person differences in the relationships between psychological distress and productivity loss. For the alternative measure of presenteeism, we mainly fitted Poisson regression. We fitted the Negative binomial regression technique as an alternative regression technique to check if the results hold. To ensure the robustness of our results, we also performed additional analyses by incorporating a new variable (chronic conditions) into the main regression models to account for potential confounding factors.

#### Heterogenous effects

While we have examined the average effects of psychological distress on productivity loss, the extent to which this effects varies across different population subgroups remains unclear. To explore how the relationship between psychological distress and productivity loss differed across demographic groups, we conducted stratified analyses by age groups and sexes following a prior study [43]. We have applied the same regression models as in our primary analysis. More specifically, we used longitudinal Fixed-effects regression for sickness absence, presenteeism, and underemployment, and applied Poisson regression for the alternative measure of presenteeism.

#### Estimates of psychological distress-attributable costs of sickness absence and presenteeism

We calculated the average annual costs of sickness absence and presenteeism attributable to moderate and high levels of psychological distress among Australian working adults. To estimate the costs of sickness absence, we employed the following formula following an earlier study [44].

Sickness absence costs = (Number of days missed in the past 12 months) x (Average daily gross wages and salary).

We have estimated the number of days missed in the past 12 months in two ways. Firstly, we considered the mean difference in sick leave days by psychological distress without adjusting covariates (please refer to Appendix Table A4). Secondly, we have calculated the average marginal (partial) effects of moderate and high psychological distress considering zero random effects. We have calculated the average daily gross wages and salary in two ways (please refer to Appendix Table A5). Firstly, we calculated the mean wages and salaries from the study sample. Secondly, we collected gross wages and salaries data from the Australian Bureau of Statistics.

We have also estimated psychological distress-attributable costs of presenteeism among working adults in Australia using the following formula.

Presenteeism costs = (Number of days worked despite feeling unwell in the past 4 weeks) x 13 x (% of work/other activities have been accomplished as carefully as usual in a day when working despite being unwell x (Average daily gross wages and salary). The survey collected data on the number of days participants worked while unwell during the preceding four weeks. To estimate annual presenteeism, we multiplied the average number of days worked despite illness in the past four weeks by 13, assuming a consistent pattern of presenteeism throughout the year.

To estimate presenteeism, we calculated the number of days worked while unwell in the past four weeks using a similar methodology to that employed for sickness absence. We assumed that a working adult had accomplished 50% of work/other activities as carefully as usual in a day when working despite being unwell. For sensitivity analyses, we assumed that a working adult had accomplished 40%, or 60%, of work/other activities as carefully as usual in a day when working despite being unwell (please refer to Appendix Table A6). We multiplied the additional number of days worked despite feeling unwell by 13 to get the value for the previous 12 months.

## Results

### Descriptive analyses

Table 1 shows the baseline, final, and pooled socio-demographic, health, and job-related characteristics of the analytical sample. Our study comprised a total of 70,973 observations from 18,729 working adults, of which most of them (67%) were aged 25 to 54 years. In the pooled data, half of the samples were male (50%), nearly two-thirds were partnered (65%), approximately one-third had university qualifications (32%), mostly non-Indigenous (98%), and resided in major cities (69%). Table 1 also revealed that around 23% of participants were living with obesity, 18% were living with a disability, 18% currently smoke, 88% consumed alcohol, and 65% performed less than the recommended level of physical activity. The results further showed that the majority of respondents were employed in small firms (43%), held permanent positions (74%), and were professionals (25%). Most did not hold supervisory roles (56%), were not union members (77%), and had access to both paid holiday leave (79%) and sick leave (80%). The mean job satisfaction score of the working adults was 7.69 (out of 10), with a standard deviation of 1.58 (pooled data).

**Table 1:** Distribution of the analytic sample (socio-demographic, health, and job-related characteristics): baseline, final, and pooled data

Variables	Baseline (Wave 7)		Final timepoint (Wave 21)		Pooled data*	
	*n*	%	*n*	%	*n*	%
Socio-demographic characteristics						
Age						
17–24 years	1,206	17.14	1,251	13.50	11,123	15.67
25–54 years	4,864	69.11	6,241	67.37	47,607	67.08
55–67 years	968	13.75	1,772	19.13	12,243	17.25
Sex						
Male	3,580	50.87	4,503	48.61	35,773	50.40
Female	3,458	49.13	4,761	51.39	35,200	49.60
Relationship status						
Partnered	4,485	63.73	6,079	65.62	45,875	64.64
Unpartnered	2,553	36.27	3,185	34.38	25,098	35.36
Highest education level completed						
Year 12 and below	2,845	40.42	2,633	28.42	23,638	33.31
Professional qualifications	2,267	32.21	3,189	34.42	24,406	34.39
University qualifications	1,926	27.37	3,442	37.15	22,929	32.31
Household yearly disposable income						
Quintile 1 (Poorest)	1,408	20.01	1,853	20.00	14,195	20.00
Quintile 2 (Poorer)	1,408	20.01	1,853	20.00	14,195	20.00
Quintile 3 (Middle)	1,408	20.01	1,853	20.00	14,196	20.00
Quintile 4 (Richer)	1,407	19.99	1,853	20.00	14,193	20.00
Quintile 5 (Richest)	1,407	19.99	1,852	19.99	14,194	20.00
Indigenous status						
Not of Indigenous origin	6,925	98.39	9,017	97.33	69,538	97.98
Indigenous origin	113	1.61	247	2.67	1,435	2.02
Region of residence						
Major city	4,728	67.18	6,356	68.61	49,125	69.22
Regional city	2,182	31.00	2,798	30.20	20,842	29.37
Remote area	128	1.82	110	1.19	1,006	1.42
Health-related characteristics						
BMI						
Underweight	138	1.96	130	1.40	1,265	1.78
Healthy weight	2,925	41.56	3,261	35.20	27,449	38.68
Overweight	2,525	35.88	3,354	36.20	25,607	36.08
Obesity	1,450	20.60	2,519	27.19	16,652	23.46
Long-term health condition / Disability						
No	5,877	83.50	7,493	80.88	58,441	82.34
Yes	1,161	16.50	1,771	19.12	12,532	17.66
Health-related behaviours						
Smoking status						
Non-smoker	5,413	76.91	7,876	85.02	58,009	81.73
Current smoker	1,625	23.09	1,388	14.98	12,964	18.27
Alcohol consumption						
Non-drinker	722	10.26	1,232	13.30	8,629	12.16
Current drinker	6,316	89.74	8,032	86.70	62,344	87.84
Physical activity						
Less than the recommended level	4,606	65.44	5,950	64.23	46,116	64.98
Recommended level	2,432	34.56	3,314	35.77	24,857	35.02
Job-related characteristics						
Firm size						
Small (1–19 employees)	3,131	44.49	3,779	40.79	30,283	42.67
Medium (20–99 employees)	1,916	27.22	2,611	28.18	19,369	27.29
Large (≥ 100 employees)	1,991	28.29	2,874	31.02	21,321	30.04
Employment contract						
Permanent	5,157	73.27	7,184	77.55	52,175	73.51
Fixed-term	587	8.34	655	7.07	6,139	8.65
Casual	1,294	18.39	1,425	15.38	12,659	17.84
Occupation						
Professional	1,704	24.21	2,573	27.77	18,120	25.53
Manager	874	12.42	1,307	14.11	9,613	13.54
Technicians and trade workers	982	13.95	1,126	12.15	9,304	13.11
Community and personal service workers	759	10.78	1,122	12.11	8,262	11.64
Clerical and administrative workers	1,090	15.49	1,196	12.91	10,046	14.15
Sales workers	628	8.92	692	7.47	5,645	7.95
Machinery operators and drivers	400	5.68	583	6.29	4,231	5.96
Labourers	601	8.54	665	7.18	5,752	8.10
Supervisory responsibilities						
Yes	3,328	47.29	3,903	42.13	32,086	45.21
No	3,710	52.71	5,361	57.87	38,887	54.79
Union membership						
Yes	1,684	23.93	1,973	21.30	16,517	23.27
No	5,354	76.07	7,291	78.70	54,456	76.73
Paid holiday leave						
Yes	5,520	78.43	7,665	82.74	56,293	79.32
No	1,518	21.57	1,599	17.26	14,680	20.68
Paid sick leave						
Yes	5,528	78.55	7,687	82.98	56,458	79.55
No	1,510	21.45	1,577	17.02	14,515	20.45
Overall job satisfaction, mean (SD)	7,038	7.69 (1.65)	9,264	7.90 (1.43)	70,973	7.69 (1.58)

Notes: (1) * Indicates that every two waves from 2007 to 2021 were pooled. (2) We used a ‘modified OECD’ equivalence scale to measure equivalised household annual disposable income. (3) For pooled data, unique persons = 19,145; yearly observations = 72,575)

The distribution of the outcome variables (sickness absence, presenteeism, and underemployment) and the key variables of interest (psychological distress) at the baseline (2007), final timepoint (2021), and pooled data were presented in Table 2. The results showed that the mean sickness absence in the pooled data was 2.90 days, with a high standard deviation of 6.59 days. The results further showed that more than one-fifth of the respondents (21%) reported presenteeism, and a significant proportion of the sample (9%) experienced underemployment. In this study, we used an alternative measure of presenteeism. The results showed that the average number of days worked despite feeling either physically or mentally unwell during the past 4 weeks among the study participants was 1.48 days (SD 3.83). According to Tables 2 and 15%, 22%, and 63% of working adults reported high, moderate, and low levels of psychological distress, respectively (pooled data).

**Table 2:** Distribution of the outcome and main variables of interest: baseline, final, and pooled data (unique persons = 18,729; yearly observations = 70,973)

Variables	Baseline (Wave 7)		Final timepoint (Wave 21)		Pooled data*	
	*n*	%	*n*	%	*n*	%
Outcome variables						
Presenteeism						
No	5,747	81.66	6,629	71.56	56,117	79.07
Yes	1,291	18.34	2,635	28.44	14,856	20.93
Alternative measure of presenteeism						
Number of days worked despite feeling physically or mentally unwell during the past 4 weeks (mean [SD])	—	—	9,264	1.48 (3.83)	—	—
Days of sick leave taken in the past 12 months (mean [SD])	7,038	2.61 (5.21)	9,264	2.99 (6.58)	70,973	2.90 (6.59)
Underemployment						
No	6,497	92.31	8,551	92.30	64,544	90.94
Yes	541	7.69	713	7.70	6,429	9.06
Main variable of interest						
Psychological distress						
Low	4,674	66.41	4,691	50.64	44,499	62.70
Moderate	1,521	21.61	2,432	26.25	15,707	22.13
High	843	11.98	2,141	23.11	10,767	15.17

Notes: (1) * Indicates that every two waves from 2007 to 2021 were pooled. (2) Decimal numbers for sickness absence were rounded to the value 1. (3) Values are rounded off to two decimal places. (4) Data on alternative measures of presenteeism were available only for wave 21

**Fig. 2:**
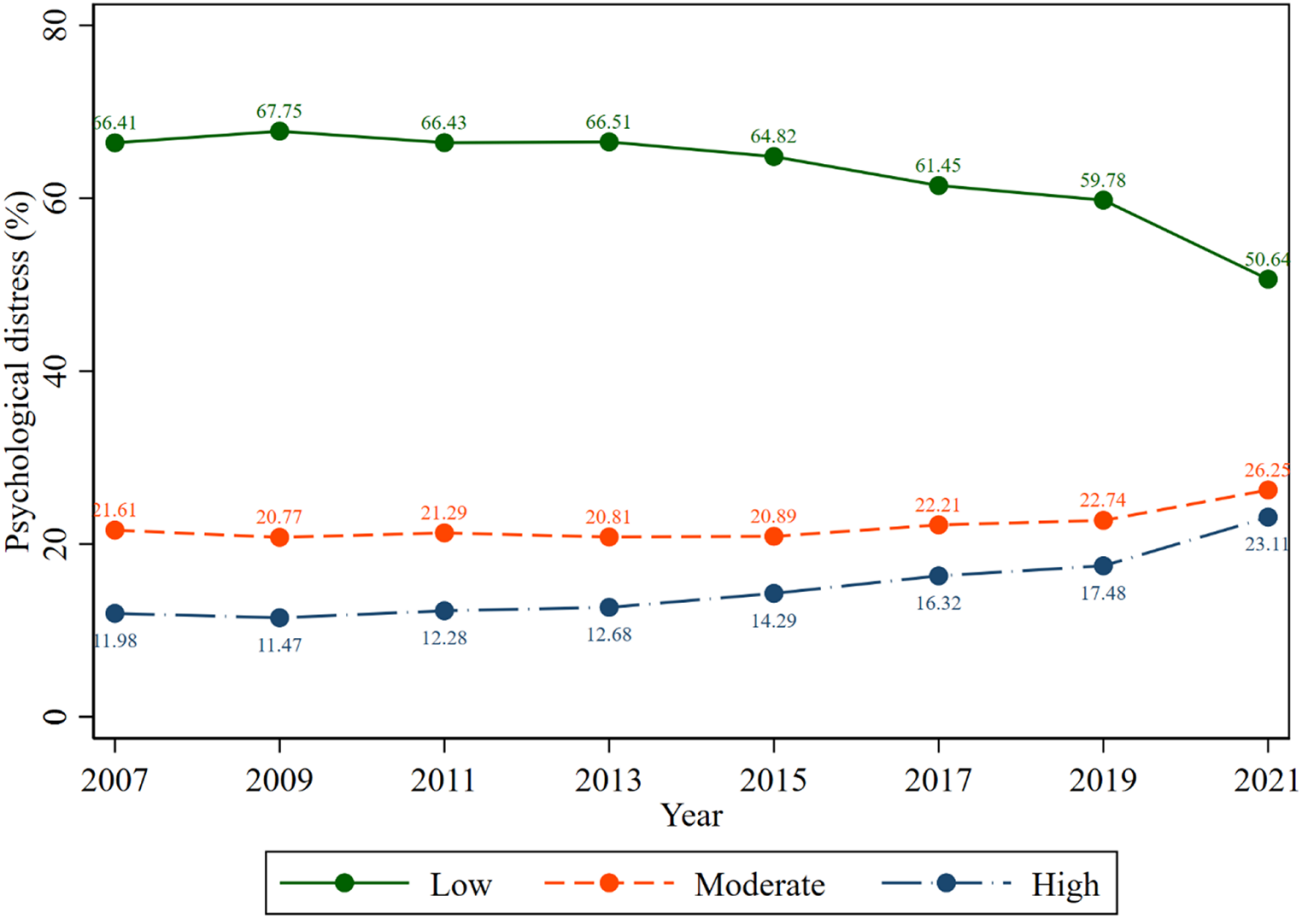
Trend in the proportion of psychological distress

Figure 2 depicts the proportion of Australian workers experiencing psychological distress during the study period (every other year from 2007 to 2021). The figure showed that the proportion of moderate and high psychological distress was increasing among Australian working adults. The proportion of high psychological distress rose over the study period, ranging from 11.47% (2009) to 23.11% (2021). In a similar fashion, moderate psychological distress has risen from 20.89% (2015) to 26.25% (2021).

**Fig. 3:**
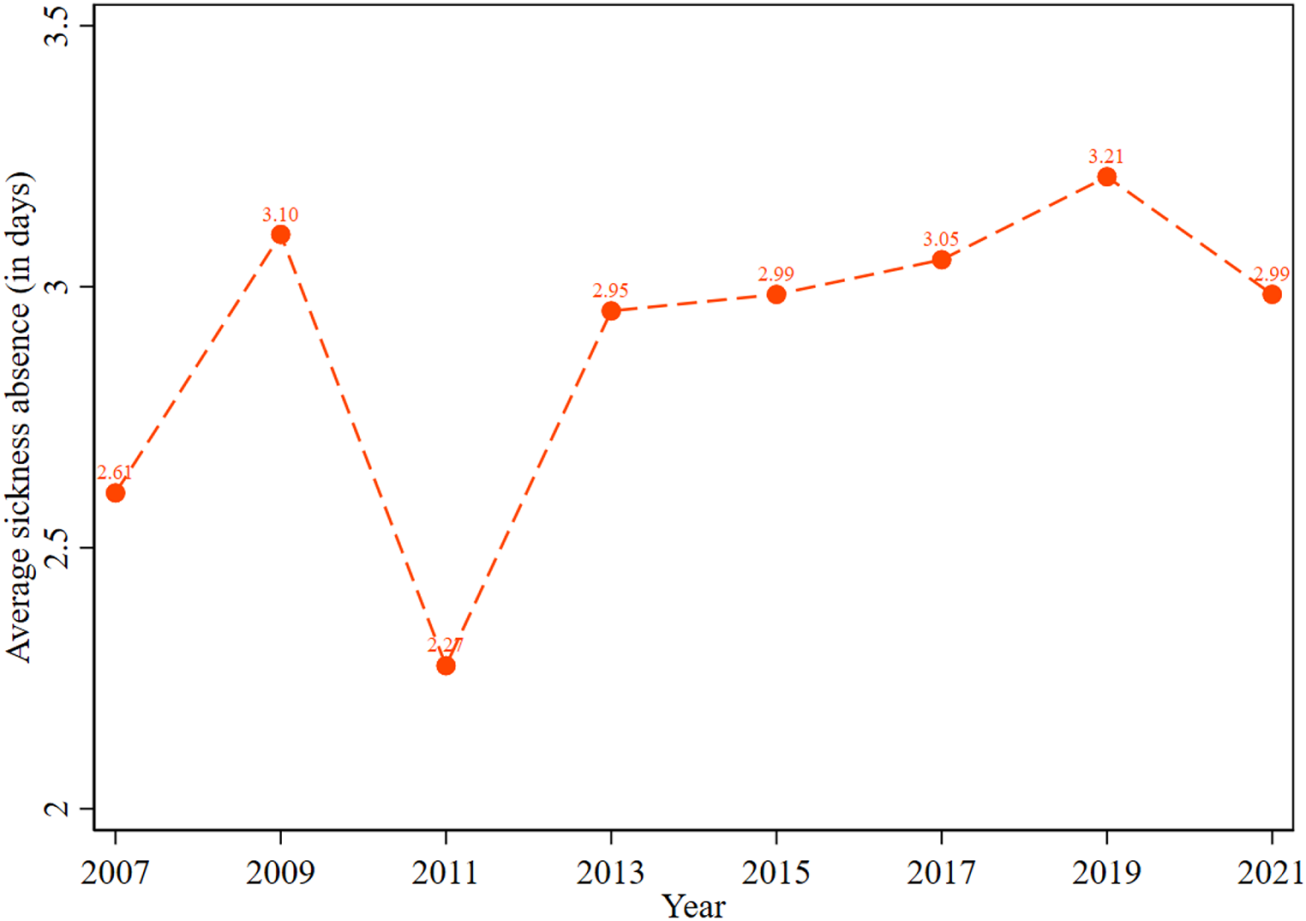
Trend of the average sick leave days including zero

Figure 3 displays the trend in mean sickness absence, including zero. The figure showed that mean sickness absence ranges from 2.61 days (2007) to 3.21 days (2019) per year. The figure also showed that average sickness days were approximately 3 days over the study period.

**Fig. 4:**
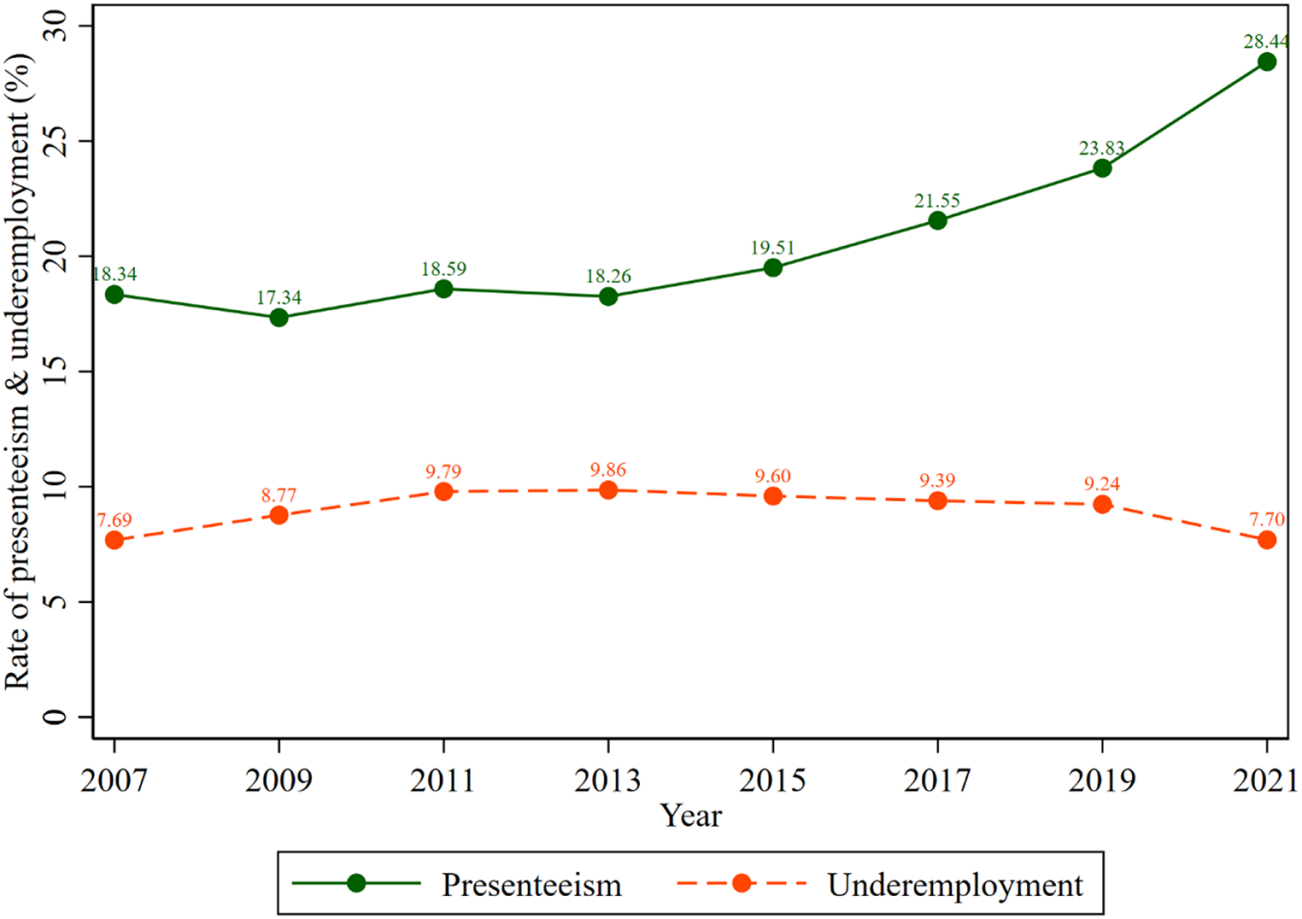
Trend in the proportion of presenteeism and underemployment

Figure 4 illustrates the trend in the proportion of presenteeism and underemployment among Australian working adults. The figure showed that the proportion of presenteeism has increased over the study periods. The proportion of presenteeism has increased from 17.34% (2009) to 28.44% (2021). However, the proportion of underemployment oscillated between 7.69% (2007) to 9.79% (2011).

**Fig. 5:**
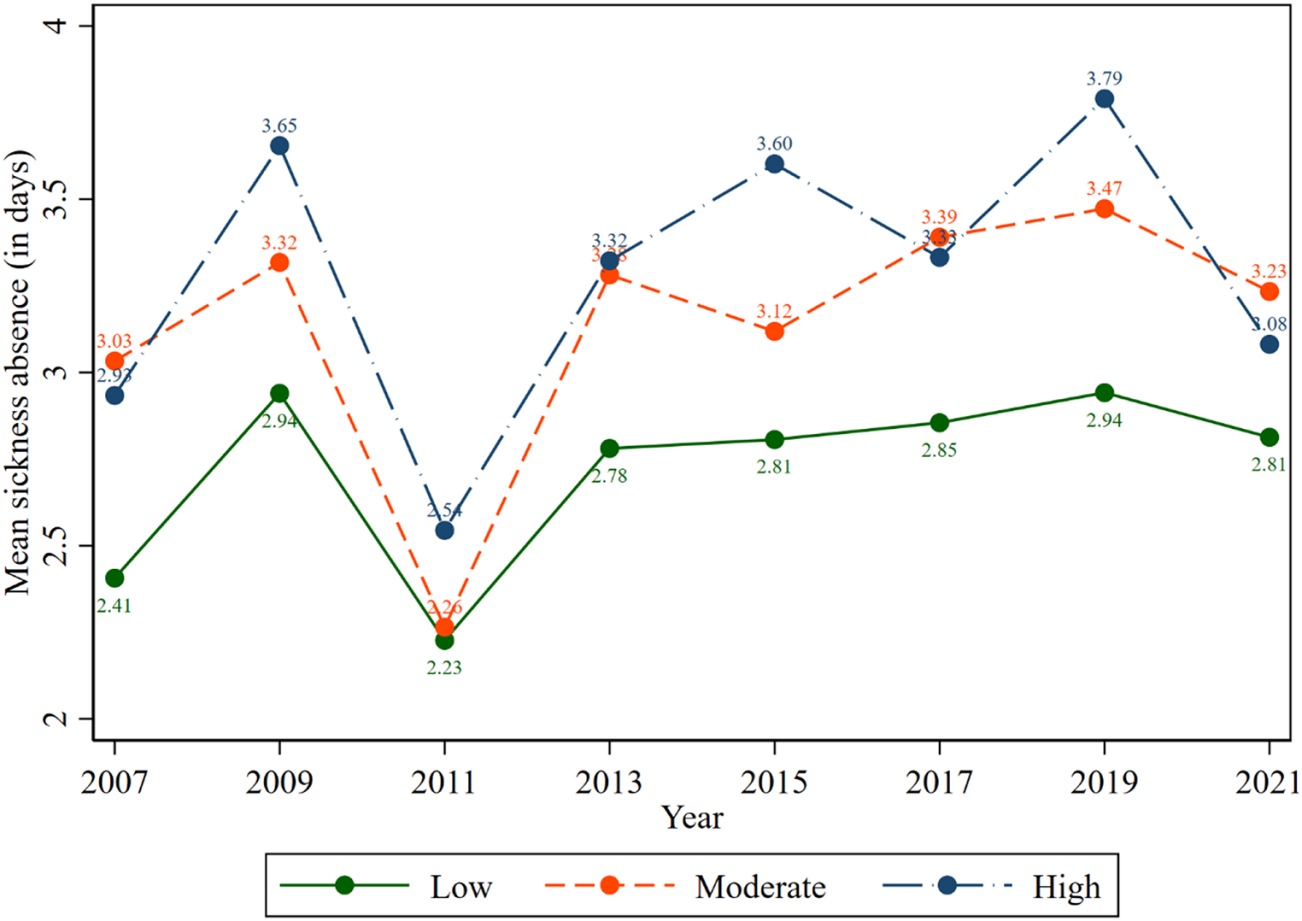
Trend of the average sick leave days, including zero over psychological distress

Figure 5 depicts the trend of the mean sickness absence days of the study participants by their psychological distress level. It is observed that working adults with moderate and high psychological distress took higher sickness absences over the study period compared to peers with low psychological distress. For example, the average sickness absence (including zero) among working adults with low, moderate, and high psychological distress were 2.81, 3.23, and 3.08 days, respectively (2021).

**Fig. 6:**
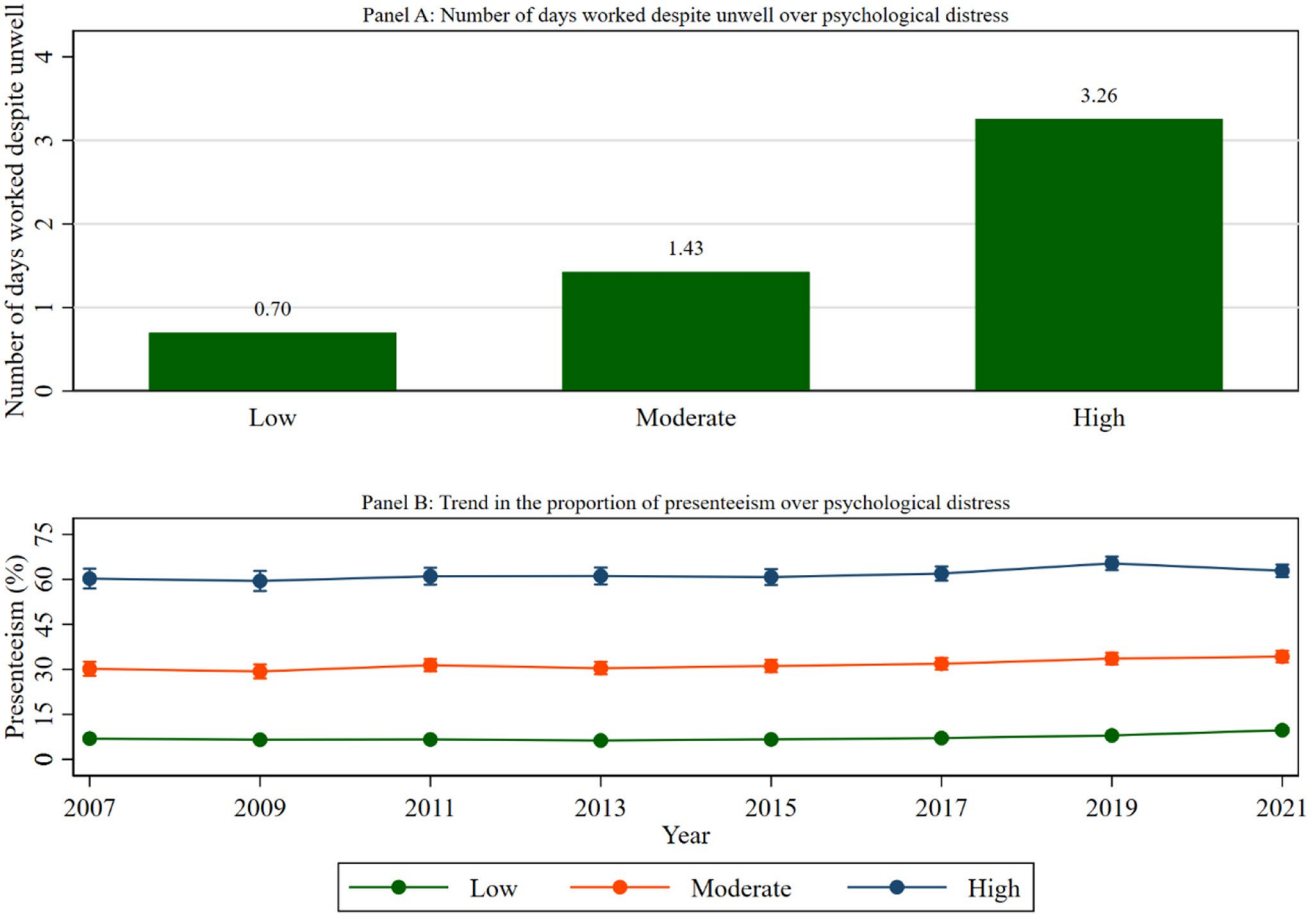
Presenteeism over psychological distress

Figure 6 shows the rate of presenteeism according to the psychological distress status of the study participants. The figure (Panel A) showed that average days worked despite feeling either physically or mentally unwell during the past 4 weeks over psychological distress in 2021. It is observed that average worked days despite feeling unwell were highest for people living with high psychological distress (3.26) followed by moderate (1.43) and low psychological distress (0.70).

The figure (Panel B) also indicated that working adults with high psychological distress had the highest rate of presenteeism, following moderate and low psychological distress in all studied waves. For example, the proportion of presenteeism among the workers with high psychological distress was highest (62.87%), compared to peers with moderate (34.25%) and low psychological distress (9.72%) in 2021.

**Fig. 7:**
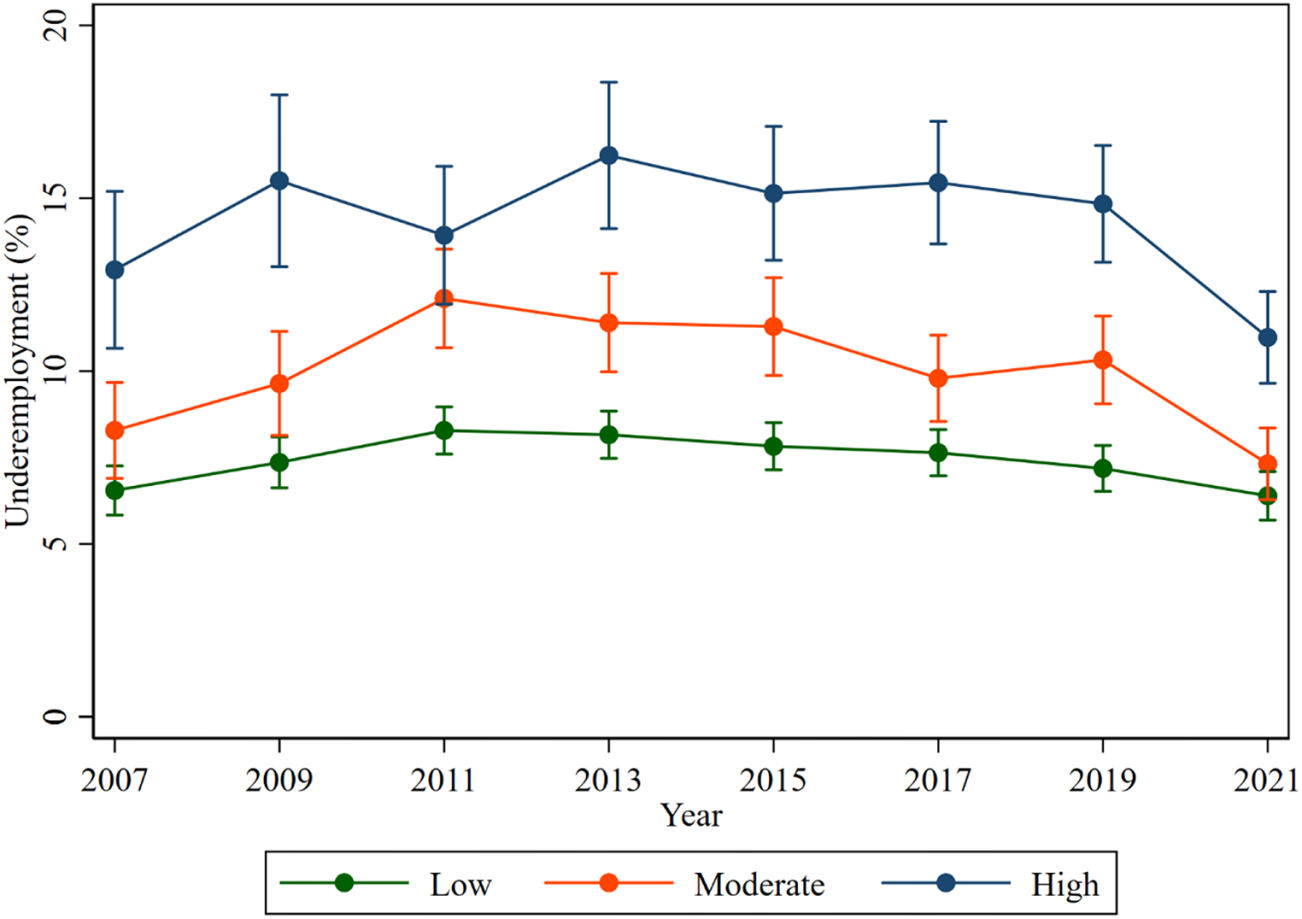
Proportion of underemployment over psychological distress

Figure 7 demonstrates the trend in the proportion of underemployment by psychological distress. The proportion of underemployment was highest among workers with high psychological distress in all studied waves. For example, the rate of underemployment among workers with high psychological distress was 10.98%, followed by moderate (7.32%) and low (6.40%) psychological distress, respectively, in 2021.

Table 3 displays the transition rates (moving from low to high) of each type of psychological distress. The rows represent values at different categories of psychological distress during the baseline timepoint, and the columns indicate the values at subsequent timepoints. Table 3 showed that 80.70% (27,492 observations), 39.64% (4,460 observations), and 53.96% (3,738 observations) of the sample with low, moderate, and high psychological distress, respectively, at the baseline, remained in the same condition in the subsequent timepoints.

Approximately, 14.72% and 4.58% of working adults with low psychological distress at baseline transitioned to moderate and high psychological distress, respectively, throughout the study period. Similarly, of working adults who initially experienced moderate psychological distress, 39.96% transitioned to a state of low psychological distress, while 20.41% developed high psychological distress. Finally, it is found that within the set of observations displaying high psychological distress, 17.93% and 28.11% moved to a state of low and moderate psychological distress, respectively.

**Table 3:** Estimated transition rate for each level of psychological distress (from T to T + 1+…+n)

Psychological distress	Psychological distress			Total
	Low, *n* (%)	Moderate, *n* (%)	High, *n* (%)	
Low	27,492 (80.70)	5,013 (14.72)	1,560 (4.58)	34,065 (100)
Moderate	4,496 (39.96)	4,460 (39.64)	2,296 (20.41)	11,252 (100)
High	1,242 (17.93)	1,947 (28.11)	3,738 (53.96)	6,927 (100)
**Total**	33,230 (63.61)	11,420 (21.86)	7,594 (14.54)	52,244 (100)

Notes:1. T indicates the timepoint. 2. The total number of yearly observations used to calculate the transition rate is 52,244

### Regression modelling: psychological distress and productivity loss (sickness absence, presenteeism, and underemployment)

Table 2 provides the abridged regression results of the effects of psychological distress on productivity in the workplace, as measured by sickness absence, presenteeism, and underemployment. Complete regression results, including all covariates and detailed estimates, are available in Appendix Tables A2 and A3. According to the findings of the longitudinal Fixed-effects Poisson regression (Model 1), moderate (IRR: 1.08, 95% CI: 1.06, 1.10) and high (IRR: 1.13, 95% CI: 1.11, 1.16) psychological distress were associated with a higher rate of sickness absence (Table 4). The result also showed that when all other covariates remained constant, working adults with moderate (OR: 4.75, 95% CI: 4.39, 5.15) and high (OR: 19.24, 95% CI: 17.26, 21.45) psychological distress were more likely to report presenteeism when they moved from low psychological distress (Model 2). As an alternative measure of presenteeism, we examined the number of days a working adult worked while unwell, providing insights into the effects of psychological distress on work performance. Our study found that a working adult with moderate (IRR: 1.85, 95% CI: 1.77, 1.95) and high psychological distress (IRR: 3.69, 95% CI: 3.53, 3.86) has a higher rate of working a greater number of days while unwell compared to those with low psychological distress (Model 3). These findings suggest that psychological distress can lead to increased presenteeism, where individuals continue to work despite illness, potentially compromising productivity. However, we did not find any statistically significant relationship between psychological distress and underemployment (Model 4).

**Table 4:** The effects of psychological distress on absenteeism, presenteeism, and underemployment, abridged results

Variables	Sickness absence	Presenteeism	An alternative measure of presenteeism	Underemployment
	Model 1: FE Poisson regression	Model 2: FE logistic regression	Model 3: Poisson regression	Model 4: FE logistic regression
	aIRR (95% CI)	aOR (95% CI)	aIRR (95% CI)	aOR (95% CI)
**Psychological Distress**				
Low (ref)				
Moderate	1.08***	4.75***	1.85***	1.08
	[1.06–1.10]	[4.39–5.15]	[1.77–1.95]	[0.97–1.20]
High	1.13***	19.24***	3.69***	1.10
	[1.11–1.16]	[17.26–21.45]	[3.53–3.86]	[0.97–1.26]
Observations	n_observations_ = 52,314	n_observations_ = 31,825	—	n_observations_ = 17,143
	n_individuals_ = 10,376	n_individuals_ = 6,067	n_individuals_ = 9,264	n_individuals_ = 3,461
Individual fixed effects	Yes	Yes		Yes
Wave fixed effects	Yes	Yes		Yes

Notes: (1) 95% confidence intervals are reported in parentheses. (2) ***, **, and * denote significance at the *p* < 0.01,*p* < 0.05, and *p* < 0.1 levels, respectively. (3) Abbreviations: FE = Fixed-effects; aOR = Adjusted Odds Ratio; aIRR = Adjusted Incidence Rate Ratio; Ref = reference category. (4) values are rounded off to two decimal places. (5) Values in bold are statistically significant. (6) All models were adjusted for age, relationship status, highest education level completed, household yearly disposable income, region of residence, BMI, disability, smoking status, alcohol consumption, physical activity, firm size, employment contract, occupation, industry, supervisory responsibilities, union membership, paid holiday leave, paid sick leave, and overall job satisfaction

### Robustness checks

#### Alternative estimator

The main goal of our study is to identify the within-person differences in the relationships between psychological distress and productivity loss. Therefore, we used Fixed-effects regression modelling. However, we also fitted Random-effects regression modelling to identify the between-person differences in the relationships between psychological distress and productivity loss. Results from Random-effects Poisson regression, and Random-effects logistic regression showed that moderate and high psychological distress were statistically significant for sickness absence, presenteeism, and underemployment (Table 5). Additionally, we fitted Negative binomial regression for the number of days a working adult worked while unwell (an alternative measure of presenteeism) (Model 3). The results from the Random-effects model were consistent with the Fixed-effects model, indicating that the relationship between psychological distress and productivity loss (sickness absence and presenteeism) was robust (Models 1, 2, & 3). While the Fixed-effects model did not find a significant association between psychological distress and underemployment, the Random-effects model revealed a positive relationship. The results demonstrated that the likelihood of underemployment is considerably higher among working adults with moderate (aOR: 1.19, 95% CI: 1.10–1.29) and high (aOR: 1.47, 95% CI: 1.33–1.61) psychological distress compared to the counterparts with low psychological distress (Model 4).

**Table 5:** The relationships between psychological distress with sickness absence, presenteeism, and underemployment

Variables	Sickness absence	Presenteeism	An alternative measure of presenteeism	Underemployment
	Model 1: RE Poisson regression	Model 2: RE logistic regression	Model 3: Negative binomial regression	Model 4: RE logistic regression
	aIRR (95% CI)	aOR (95% CI)	aIRR (95% CI)	aOR (95% CI)
**Psychological Distress**				
Low (ref)				
Moderate	1.09***	6.80***	1.86***	1.19***
	[1.07–1.10]	[6.37–7.25]	[1.62–2.13]	[1.10–1.29]
High	1.14***	31.58***	4.02***	1.47***
	[1.12–1.17]	[29.10–34.28]	[3.48–4.65]	[1.33–1.61]
Observations	n_observations_ = 70,973	n_observations_ = 70,973	—	n_observations_ = 70,973
	n_individuals_ = 18,729	n_individuals_ = 18,729	n_individuals_ = 9,264	n_individuals_ = 18,729

Notes: (1) Abbreviations: RE = Random-effects; aOR = Adjusted Odds Ratio; aIRR = Adjusted Incidence Rate Ratio; Ref = reference category. (2) All models were adjusted for age, gender, relationship status, highest education level completed, household yearly disposable income, Indigenous status, region of residence, BMI, disability, smoking status, alcohol consumption, physical activity, firm size, employment contract, occupation, industry, supervisory responsibilities, union membership, paid holiday leave, paid sick leave, and overall job satisfaction. (3) For other details, please see notes 1,2,4, and 5 in Table 4

#### Incorporate chronic conditions in the regression model

To assess the robustness of our findings, we conducted sensitivity analyses by including an additional variable in the regression model. We performed robustness checks by incorporating chronic conditions in the regression model (Table 6). The reason is a chronic condition poses a greater risk of productivity loss at work by increasing sickness absence, presenteeism, and underemployment. Data on chronic conditions is not available on all the studied waves. Therefore, the chronic condition was not included in the main regression model earlier. The inclusion of chronic conditions as an additional covariate did not alter our main findings (Table 6). We observed that key results for our main exposure variable, psychological distress, were similar in magnitude and significance after adding chronic condition to the main regression model. The results from the longitudinal Fixed-effects Poisson and logistic regression models demonstrated the likelihood of higher sickness absence, and presenteeism linked with moderate and high levels of psychological distress (Models 1, 2, & 3). Additionally, we did not find evidence to suggest that psychological distress is associated with underemployment (Model 4).

**Table 6:** The effects of psychological distress on absenteeism, presenteeism, and underemployment

Variables	Sickness absence	Presenteeism	An alternative measure of presenteeism	Underemployment
	Model 1: FE Poisson regression	Model 2: FE logistic regression	Model 3: Poisson regression	Model 4: FE logistic regression
	aIRR (95% CI)	aOR (95% CI)	aIRR (95% CI)	aOR (95% CI)
**Psychological Distress**				
Low (ref)				
Moderate	1.07***	4.75***	1.79***	1.07
	[1.06–1.09]	[4.38–5.14]	[1.71–1.88]	[0.97–1.19]
High	1.12***	19.09***	3.33***	1.09
	[1.10–1.14]	[17.12–21.28]	[3.18–3.49]	[0.95–1.25]
**Chronic conditions**				
No morbidity (ref)				
Single chronic condition	1.11***	1.18**	1.50***	1.04
	[1.09–1.13]	[1.06–1.30]	[1.44–1.56]	[0.91–1.18]
Multimorbidity	1.17***	1.19*	1.89***	1.35**
	[1.15–1.20]	[1.03–1.36]	[1.80–1.98]	[1.12–1.62]
Observations	n_observations_ = 52,314	n_observations_ = 31,285	—	n_observations_ = 17,143
	n_individuals_ = 10,376	n_individuals_ = 6,067	n_individuals_ = 9,264	n_individuals_ = 3,461
Individual fixed effects	Yes	Yes		Yes
Wave fixed effects	Yes	Yes		Yes

Notes: (1) We utilised waves 9, 13, 17, and 21 to incorporate chronic conditions in the regression models. (2) For other details, please see notes 1–6 in Table 4. (3) Abbreviations: FE = Fixed-effects; aOR = Adjusted Odds Ratio; aIRR = Adjusted Incidence Rate Ratio; Ref = reference category

### Heterogenous effects of psychological distress on productivity

The presence of heterogeneity in the sample can mask true relationships and lead to misleading conclusions. Therefore, it is important to consider potential subgroup differences when interpreting the results. Table 7 showed the effects of psychological distress on sickness absence, presenteeism, and underemployment by age group and sex. Our analysis revealed that moderate and high psychological distress were associated with increased rates of sickness absence across all age groups and sexes (Panel A: Models 1, 2, 4, & 5), except for individuals aged 17–24 years (Panel A: Model 3). Our subgroup analyses by age and sex also reinforced the main findings that individuals with moderate and high psychological distress were more likely to experience presenteeism (Panel B: Models 1–5). The findings held consistent when the regression models were repeated using an alternative measure of presenteeism (Panel C: Models 1–5). While the main analysis did not find a significant association between psychological distress and underemployment, subgroup analysis revealed that individuals aged 25–54 years with high psychological distress had 1.23 times higher odds (OR: 1.23, 95% CI: 1.03, 1.46) to be underemployed (Panel D: Model 4).

**Table 7:** The effects of psychological distress on absenteeism, presenteeism, and underemployment, stratified by age and sex

Panel A — Sickness Absence					
Variables	By sex		By age groups		
	Model 1: Male	Model 2: Female	Model 3: 17–24 years	Model 4: 25–54 years	Model 5: 55–67 years
	**aIRR (95% CI)**	**aIRR (95% CI)**	**aIRR (95% CI)**	**aIRR (95% CI)**	**aIRR (95% CI)**
**Psychological distress**					
Low (ref)					
Moderate	1.08***	1.08***	0.99	1.05***	1.14***
	[1.06–1.10]	[1.06–1.10]	[0.93–1.06]	[1.03–1.07]	[1.09–1.18]
High	1.11***	1.15***	1.04	1.12***	1.33***
	[1.08–1.15]	[1.12–1.18]	[0.96–1.12]	[1.09–1.14]	[1.24–1.42]
Observations	n_observations_ = 25,717	n_observations_ = 26,595	n_observations_ = 5,259	n_observations_ = 35,511	n_observations_ = 7,017
	n_individuals_ = 5,015	n_individuals_ = 5,360	n_individuals_ = 1,880	n_individuals_ = 7,733	n_individuals_ = 1,979
Individual fixed effects	Yes	Yes	Yes	Yes	Yes
Wave fixed effects	Yes	Yes	Yes	Yes	Yes
**Panel B — Presenteeism**					
**Variables**	**By sex**		**By age groups**		
	**Model 1: Male**	**Model 2: Female**	**Model 3: 17–24 years**	**Model 4: 25–54 years**	**Model 5: 55–67 years**
	**aOR (95% CI)**	**aOR (95% CI)**	**aOR (95% CI)**	**aOR (95% CI)**	**aOR (95% CI)**
**Psychological distress**					
Low (ref)					
Moderate	4.16***	5.30***	5.07***	4.76***	4.48***
	[3.70–4.68]	[4.75–5.91]	[3.83–6.71]	[4.31–5.26]	[3.58–5.60]
High	15.73***	22.92***	20.33***	19.50***	15.40***
	[13.41–18.43]	[19.74–26.62]	[14.31–28.87]	[17.01–22.34]	[10.82–21.91]
Observations	n_observations_ = 14,268	n_observations_ = 17,016	n_observations_ = 2,877	n_observations_ = 19,824	n_observations_ = 3,721
	n_individuals_ = 2,736	n_individuals_ = 3,331	n_individuals_ = 998	n_individuals_ = 4,156	n_individuals_ = 986
Individual fixed effects	Yes	Yes	Yes	Yes	Yes
Wave fixed effects	Yes	Yes	Yes	Yes	Yes
**Panel C — Number of days worked despite feeling physically or mentally unwell (Alternative measure of presenteeism)**					
**Variables**	**By sex**		**By age groups**		
	**Model 1: Male**	**Model 2: Female**	**Model 3: 17–24 years**	**Model 4: 25–54 years**	**Model 5: 55–67 years**
	**aIRR (95% CI)**	**aIRR (95% CI)**	**aIRR (95% CI)**	**aIRR (95% CI)**	**aIRR (95% CI)**
**Psychological distress**					
Low (ref)					
Moderate	1.71***	2.00***	1.84***	1.80***	2.01***
	[1.59–1.83]	[1.87–2.14]	[1.56–2.18]	[1.70–1.91]	[1.81–2.23]
High	3.37***	3.86***	4.18***	3.61***	3.65***
	[3.16–3.60]	[3.62–4.11]	[3.61–4.84]	[3.42–3.81]	[3.28–4.06]
Observations	n_observations_ = 4,503	n_observations_ = 4,761	n_observations_ = 1,251	n_observations_ = 6,241	n_observations_ = 1,772
**Panel D — Underemployemnt**					
**Variables**	**By sex**		**By age groups**		
	**Model 1: Male**	**Model 2: Female**	**Model 3: 17–24 years**	**Model 4: 25–54 years**	**Model 5: 55–67 years**
	**aOR (95% CI)**	**aOR (95% CI)**	**aOR (95% CI)**	**aOR (95% CI)**	**aOR (95% CI)**
**Psychological distress**					
Low (ref)					
Moderate	1.06	1.09	1.01	1.09	1.08
	[0.93–1.22]	[0.93–1.28]	[0.79–1.30]	[0.95–1.24]	[0.74–1.57]
High	1.09	1.14	0.83	1.23*	1.25
	[0.91–1.30]	[0.94–1.40]	[0.62–1.12]	[1.03–1.46]	[0.72–2.17]
Observations	n_observations_ = 9,651	n_observations_ = 7,490	n_observations_ = 2,562	n_observations_ = 9,986	n_observations_ = 1,379
	n_individuals_ = 1,920	n_individuals_ = 1,540	n_individuals_ = 894	n_individuals_ = 2,132	n_individuals_ = 352
Individual fixed effects	Yes	Yes	Yes	Yes	Yes
Wave fixed effects	Yes	Yes	Yes	Yes	Yes

Notes: (1) We have fitted FE Poisson regression for all models concerning sickness absence, FE logistic regression for all models concerning presenteeism, Poisson regression for all models concerning alternative measures of presenteeism, and FE logistic regression for all models pertaining to underemployment. (2) For other details, please see notes 1–6 in Table 4

### Psychological distress-attributable costs of sickness absence and presenteeism

Table 8 provides a breakdown of the economic costs associated with psychological distress-related sickness absence and presenteeism among Australian working adults. The results showed that the costs of sickness absence due to moderate and psychological distress incurred an additional cost of AUD 60.66, and AUD 99.26 per year for a working adult in Australia (considering the average wage according to ABS). Additionally, the results indicated that males with moderate and high psychological distress incurred annual sickness absence costs of AUD 71.33 and AUD 116.73, respectively. The economic burden of psychological distress is also substantial, with moderate and high levels of distress leading to additional presenteeism costs. Our findings revealed that moderate and high psychological distress was associated with significant increases in presenteeism costs, amounting to an additional AUD 1,166.30 and AUD 3,656.05 per year, respectively, for Australian working adults (considering the average wage, according to ABS).

**Table 8:** Costs of sickness absence attributed to psychological distress according to sex

Types of productivity costs	Unit wages estimated from the HILDA sample			Unit wages, according to ABS		
	Male	Female	Overall	Male	Female	Overall
Attributable costs of yearly sickness absence due to psychological distress						
**Mean comparison**,** unadjusted**						
Moderate psychological distress	111.81	79.47	95.77	139.42	98.56	118.56
High psychological distress	150.82	107.20	129.18	188.06	132.95	159.92
**Zero random effects**,** adjusted**						
Moderate psychological distress	57.21	40.66	49.00	71.33	50.43	60.66
High psychological distress	93.61	66.54	80.18	116.73	82.52	99.26
**Attributable costs of yearly presenteeism due to psychological distress**						
**Mean comparison**,** unadjusted**						
Moderate psychological distress	1,216.94	864.96	1,042.38	1,517.44	1,072.75	1,290.37
High psychological distress	4,326.90	3,075.40	3,076.23	5,395.35	3,814.22	4,587.98
**Zero random effects**,** adjusted**						
Moderate psychological distress	1,099.93	781.79	942.15	1,371.54	969.60	1,166.30
High psychological distress	3,448.00	2,450.71	2,953.40	4,299.00	3,039.46	3,656.05

Source: Authors’ calculations

Notes: 1. Values for each cell were obtained by multiplying respective daily wages and salary with additional absent days. Please refer to Appendix Tables 4 and 5 for additional absent days, additional number of days worked despite feeling unwell, and gross wages and salary, respectively. 3. Abbreviation: ABS = Australian Bureau of Statistics. 4. All values are reported in Australian dollars and were rounded into two decimal points

## Discussion

### Key findings

We sought to examine the association between psychological distress and productivity loss through sickness absence, presenteeism, and underemployment measures among the Australian working population. Our findings revealed that working adults with moderate and high psychological distress had higher rates of sickness absence, presenteeism, and underemployment compared to the period when a working adult had low psychological distress. These findings remained significant for sickness absence, and presenteeism even after controlling for socio-demographic, health, and employment-related factors.

Consistent with findings from previous studies in Australia [11, 33, 45], we found that working adults with moderate and high psychological distress had a higher rate of work absenteeism compared to those with low psychological distress. In the UK, Hardy et al. (2003) reported that high psychological distress was significantly associated with an increased number of days absent from work [46]. The increased propensity for working adults with high psychological distress to be absent from work had been attributed to the manifest symptoms of diagnosable mental health disorders such as anxiety and depression, often associated with high levels of psychological distress [46, 47]. In other words, working adults with high psychological distress may show symptoms of diagnosable mental health disorders [48] which could compel them to go for treatment instead of work and thereby contribute to sickness absence [47]. Meanwhile, aside from the organisational costs incurred through the replacement of absent workers [13], worker absenteeism disrupts organisational functioning, especially, if no suitable replacement is available [14]. Additionally, psychological distress may intensify the risk of higher sickness absence if the available workers are compelled to take on additional responsibilities caused by the absentee worker [49]. This could contribute to further productivity loss. Therefore, our findings highlight the need for organisational managers to implement pragmatic measures that could reduce the risk of psychological distress among workers and minimise the rate of sickness absence and its adverse effects on productivity.

We also found that working adults with moderate and high psychological distress were more likely to report presenteeism relative to those with minimal psychological distress. Similar findings were reported in previous cross-sectional studies in Australia [33, 45] and Canada [18, 31].

Our study provides the first longitudinal evidence of the association between psychological distress and presenteeism among workers in Australia. Unlike individuals with physical health problems (e.g., asthma, arthritis, cardiovascular diseases, diabetes, and hypertension), persons experiencing psychological distress often show fewer symptoms and more likely to go to work despite being psychologically distressed [11, 50]. Individuals with psychological distress often contribute to productivity loss by performing less than planned, increasing the potential for workplace errors, and reducing the quality of work [50, 51]. Going to work while sick has been associated with an increased risk of more sickness episodes, prolonged illness, and a high risk of complications [52].

Our study highlights the adverse effects of high psychological distress on underemployment among working-age (25–54 years) adults. Additionally, the results from the Random-effects logistic regression showed that working adults with moderate and high psychological distress were more likely to be underemployed. Our findings are consistent with previous longitudinal studies that have demonstrated a link between psychological distress and underemployment [53–55]. In the UK, Mousteri et al. (2020) found that underemployed workers experienced high levels of psychological distress [53], which declined with the transition to full-time employment but increased with a change of job from full-time to part-time. High psychological distress among underemployed workers is often attributed to the unsatisfactory work conditions associated with underemployment, including underutilization of skills, working for a limited period, working at a lower level than actual qualification [20, 21], limited job autonomy, and lack of job security [56]. These conditions could negatively affect the feelings and emotions of underemployed workers and contribute to increased psychological distress and productivity loss [21, 54, 57]. Besides, underemployed workers are often paid low wages which do not commensurate with their expertise and level of qualifications [20]. Low income contributes to high psychological distress because it imposes difficulties in accessing basic necessities in life such as adequate food, shelter, and relaxation or leisure time activities [58]. In Finland, Virtanen et al. (2008) found that low-income earners have a 2.8 times higher risk for depression and anxiety disorders (key manifestations of psychological distress) compared to those with normal incomes [58].

### Implications for policy and practice

Despite the mounting evidence showing the greater indirect costs associated with mental health problems due to loss of productivity [2], most mental health-related policy interventions are directed at providing direct mental healthcare services, especially to persons with serious mental disorders [49]. Therefore, there is limited focus on prevention and early intervention strategies that could reduce both direct and indirect costs associated with mental disorders [49].

Despite the negative effects of psychological distress on productivity, as highlighted in our study, most organisations do not have measures in place to deal with the issue of psychological distress and associated productivity loss [13]. Our findings emphasise the need for policy interventions to reduce the risk of psychological distress and its effects on workers’ health and productivity, especially in the current global economic challenges and its associated high risk for psychological distress. Instituting interventions that improve organisational climate and minimise work-related stressors such as high effort-reward imbalance, organisational inequalities, workplace bullying, and work-life balance could significantly reduce psychological distress and limit the risk for absenteeism and presenteeism. Additionally, ensuring job security and job satisfaction, improving job autonomy, and providing alternative jobs to underemployed workers may reduce the risk of psychological distress and improve productivity. Further, instituting measures that discourage sick workers, especially those with psychological health problems, from attending work could significantly reduce the rate of presenteeism and its dire effects on productivity. Activities directed at improving worker resilience, such as encouraging support among co-workers and supervisors [59] or providing mandatory exercise programs for all workers [60] could minimize the adverse effects of psychological distress on workers’ health and productivity.

### Strengths, limitations, and avenues for further research

The major strength of our study is the use of nationally representative HILDA datasets spanning eight waves (2007 to 2021) to examine the effects of psychological distress on productivity loss measured through sickness absence, presenteeism, and underemployment. This could enhance the validity and reliability of our study and ensure the generalizability of our findings to workers in Australia. We have fitted an alternative estimator to check the relationships between psychological distress and productivity loss. The similarity in results between the Fixed-effects and Random-effects models strengthen the robustness of our findings regarding the effects of psychological distress on productivity loss. To comprehensively understand productivity loss associated with psychological distress, we employed three measures: sickness absence, presenteeism, and underemployment. Quantifying the monetary costs of presenteeism due to psychological distress can be challenging. In this study, we employed two different approaches to estimate these costs. Our study highlights the significant economic burden of psychological distress by quantifying the monetary costs associated with sickness absence and presenteeism. This information can inform policy decisions aimed at addressing mental health issues among Australian workers. However, there are some limitations inherent in our study that need to be acknowledged. Firstly, despite controlling for several demographic, health, and employment-related factors, our study could not control for work-family imbalance which is a major contributor to psychological distress among workers [60]. Secondly, since all the variables of interest (psychological distress, sickness absence, presenteeism, and underemployment) were self-reported, they were subject to recall and social desirability biases. The recall timeframe for the presenteeism variable is four weeks, and it is annual for sickness absence, and underemployment. Thirdly, while our sample size is adequate for assessing average effects, the statistical power of some subgroup analyses may be limited. Therefore, we acknowledge this potential limitation and interpret the results of subgroup analyses with caution. Fourthly, the latest round (s) of data (wave 21) were collected during COVID-19, and this may have impacted the results. Finally, available evidence suggests a significant relationship between managerial style and psychological distress [61]. However, the influence of managerial leadership on psychological distress was not accounted for in our study. Therefore, future studies should consider investigating the relationship between managerial leadership, psychological distress, and productivity loss.

## Conclusion

The rising prevalence of psychological distress among working adults is leading to substantial economic losses due to reduced productivity. Therefore, it is crucial to understand the effects of psychological distress on productivity loss. We provide evidence on the effects of psychological distress on productivity loss in the workplace. We found that psychological distress negatively affected an employee’s productivity by increasing both sickness absence and presenteeism. By highlighting the adverse effects of psychological distress on productivity, our study provides valuable insights for policymakers and healthcare professionals to develop effective strategies for prevention and treatment. To mitigate the adverse effects of psychological distress on workplace productivity, employers worldwide should consider implementing strategies to manage work-related stressors, such as workplace bullying, high effort-reward imbalance, organizational inequalities, and work-family conflict. By implementing strategies to improve job security, job autonomy, and career development opportunities, employers can create a more positive work environment and reduce the prevalence of psychological distress. By implementing these measures, employers can enhance employee productivity, reduce costs associated with psychological distress, and improve the overall quality of life of the affected individuals.

## Electronic supplementary material

Below is the link to the electronic supplementary material.

Supplementary Material 1

## Data Availability

There are two versions of the HILDA data: the General Release and the Restricted Release. This study utilised restricted release data from the Household, Income and Labour Dynamics in Australia (HILDA) Survey. Funded by the Australian Government Department of Social Services (DSS), the Survey is managed by the Melbourne Institute of Applied Economic and Social Research (MI) at the University of Melbourne. Access to the complete HILDA dataset is limited and requires specific approval, as it contains sensitive personal information. To apply for access to any of the DSS Longitudinal Studies datasets, first, all applicants and collaborators who need to view unit record data must complete and sign a once only Confidentiality Deed Poll and email the scanned, signed copy to DSS (DataAccess@dss.gov.au) and ADA (ada@ada.edu.au). Electronic signatures are currently accepted. Detailed information regarding data access procedures and requirements can be found at https://dataverse.ada.edu.au/dataverse.xhtml?alias=hilda.

## Abbreviations

OR: Odds Ratio
BMI: Body Mass Index
HILDA: Household, Income and Labour Dynamics in Australia Survey
IRR: Incidence Rate Ratio
K10: The Kessler 10 Psychological Distress Scale
SCQ: Self-completion Questionnaire

## Declarations

### Precis

Moderate to high psychological distress adversely affects a worker's job productivity through increased sickness absence, a higher risk of presenteeism, and greater underemployment.
