# Supplementary material for: Psychological distress and productivity loss: a longitudinal analysis of Australian working adults
Source: Eur J Health Econ. 2025 Apr 30;26(8):1503–24. doi: 10.1007/s10198-025-01764-9 (PMC12572030; doi:10.1007/s10198-025-01764-9)
Supplement: Supplementary file 1 — Supplementary Material 1 [file 10198_2025_1764_MOESM1_ESM.docx]

**Appendix**

Table A1: Variable list and description

| **Variables** | **Description** | **Measure** |
| --- | --- | --- |
| **Outcome variables** | | |
| Sickness absence | Days of paid sick leave in the last 12 months, including zero. | Number of days. |
| Presenteeism | Based on three questions: ‘Cut down the amount of time spent on work/other activities, Accomplished less than would like, and didn’t do work/other activities as carefully as usual.’ | 0 = No; 1 = Yes. |
| Alternative measure of presenteeism | By asking: ‘How many days during the past 4 weeks did you work despite feeling either physically or mentally unwell?’ | Number of days. |
| Underemployment | Based on two questions: ‘Hours would like to work, and Hours per week usually worked in all jobs.’ | 0 = No; 1 = Yes. |
| **Main exposure variable** | | |
| Psychological distress | Kessler Psychological Distress Scale (K10) risk categories. The K10 is a scale measuring non-specific psychological distress. | 0 = Low; 1 = Moderate; 2 =High (high, and very high). |
| **Socio-economic and demographic characteristics** | | |
| Age | Age at 30^th^ June in the year prior to the interview (in years). | 0 = 15-24 (just entering the labour market following education); 1 = 25-54 (those in their prime working lives); 2 = 55-67 (those passing the peak of their career and approaching retirement). |
| Gender | Gender of the respondent. | 0 = Male; 1 = Female. |
| Relationship status | Current marital status of the respondent. | 0 = Partnered (married in a registered marriage, and never married but living with someone in a relationship); 1 = Unpartnered (never married and not living with someone in a relationship, separated but not divorced, divorced, and widowed). |
| Highest education  level completed | Highest education level achieved by the respondent. | 0 = Year 12 and below (year 12, and year 11 and below); 1 = Professional qualifications (advanced diploma or diploma, and certificate III or IV); 2 = University qualifications (graduate diploma or certificate, bachelor or honours, postgraduate - masters or doctorate). |
| Household yearly  disposable income | Household disposable total income for the previous financial year. | 0 = Quintile 1 (poorest); 1 = Quintile 2 (poorer); 2 = Quintile 3 (middle); 3 = Quintile 4 (richer); 4 = Quintile 5 (richest). |
| Indigenous status | Aboriginal or Torres Strait Islander origin of the respondent. | 0 = Not of Indigenous origin; 1 = Indigenous origin (Aboriginal, Torres Strait Islander, and both Aboriginal and Torres Strait Islander). |
| Region of residence | Respondent’s living area by Australian Standard Geographical Classification **(**ASGS). | 0 = Major city; 1 = Regional (inner regional, and outer regional); 2 = Remote area (remote, and very remote Australia). |
| **Health-related characteristics** | | |
| BMI | BMI of the respondent, continuous. | 0 = Underweight (BMI = <18.50); 1 = Healthy weight (BMI = 18.50 to <25); 2 = Overweight (BMI = 25 to < 30); 3 = Obese (BMI = ≥ 30). |
| Long-term health condition or disability | Disability status of the respondent. | 0 = No; 1 = Yes. |
| **Health-related behavioural characteristics** | | |
| Smoking status | Based on the question: ‘Smokes cigarettes or other tobacco products?’ | 0 = Non-smoker (Never smoked, ex-smoker); 1 = Current smoker (smoke daily, smoke at least weekly, and smoke less often than weekly). |
| Alcohol consumption | Based on the question: ‘Consuming alcohol?’ | 0 = Non-drinker (never drunk, ex-drinker); 1 = Current drinker (only rarely, 1-2 days, 2-3 days, 3-4 days, 5-6 days per week and every day). |
| Physical activity | Based on the question: ‘How often do you participate in physical activity?’ | 0 = Less than the recommended level (not at all, less than once, 1 to 2, and 3 times a week); 1 = Recommended level (>3 times a week and every day). |
| **Job-related characteristics** | | |
| Firm size | Number employed at the place of work. | 0 = Small (One person, 2 to 4, 5 to 9, 10 to 19, Don’t know, but fewer than 20); 1 = Medium (20 to 49, 50 to 99, Don’t know, but 20 or more); 2 = Large (100 to 199, 200 to 499, 500 or more). |
| Employment contract | Employment contract - current job. | 0 = Permanent; 1 = Fixed-term; 2 = Casual. |
| Occupation | Occupation 1-digit ANZSCO 2006 | 0 = Professional; 1 = Manager; 2 = Technician and trade workers; 3 = Community and personal service workers; 4 = Clerical and administrative workers; 5 = Sales workers; 6 = Machinery operators and drivers; 7 = Labourers. |
| Industry |  |  |
| Supervisory responsibilities | Normally supervise the work of other employees | 0 = Yes; 1 = No. |
| Union membership | Union membership or employee association | 0 = Yes; 1 = No. |
| Paid holiday leave | Based on the question: ‘Does the employer provide paid holiday leave?’ | 0 = Yes; 1 = No. |
| Paid sick leave | Based on the question: ‘Does the employer provide paid holiday leave?’ | 0 = Yes; 1 = No. |
| Overall job satisfaction | Overall job satisfaction, 11-point scale | 0 = Totally dissatisfied; 10 = Totally satisfied. |

Notes: 1. We used a ‘modified OECD’ equivalence scale to measure equivalised household annual disposable income.

Table A2: The effects of psychological distress on sickness absence presenteeism, and underemployment (complete regression results).

| **Variables** | **Sickness absence** | | **Presenteeism** | | **Underemployment** | |
| --- | --- | --- | --- | --- | --- | --- |
|  | **Model 1: FE Poisson regression** | **Model 2: RE Poisson regression** | **Model 3: FE logistic regression** | **Model 4: RE logistic regression** | **Model 5: FE logistic regression** | **Model 6: RE logistic regression** |
|  | **aIRR (95% CI)** | **aIRR (95% CI)** | **aOR (95% CI)** | **aOR (95% CI)** | **aOR (95% CI)** | **aOR (95% CI)** |
| **Psychological Distress** |  |  |  |  |  |  |
| Low (ref) |  |  |  |  |  |  |
| Moderate | 1.08*** | 1.09*** | 4.75*** | 6.80*** | 1.08 | 1.19*** |
|  | [1.06 - 1.10] | [1.07 - 1.10] | [4.39 - 5.15] | [6.37 - 7.25] | [0.97 - 1.20] | [1.10 - 1.29] |
| High | 1.13*** | 1.14*** | 19.24*** | 31.58*** | 1.10 | 1.47*** |
|  | [1.11 - 1.16] | [1.12 - 1.17] | [17.26 - 21.45] | [29.10 - 34.28] | [0.97 - 1.26] | [1.33 - 1.61] |
| **Socio-demographic characteristics** |  |  |  |  |  |  |
| **Age** |  |  |  |  |  |  |
| 17-24 years (ref) |  |  |  |  |  |  |
| 25-54 years | 1.08*** | 1.07*** | 1.16* | 1.22*** | 0.68*** | 0.65*** |
|  | [1.05 - 1.11] | [1.04 - 1.10] | [1.00 - 1.33] | [1.12 - 1.33] | [0.58 - 0.80] | [0.58 - 0.71] |
| 55-67 years | 1.20*** | 1.17*** | 1.18 | 1.36*** | 0.52*** | 0.35*** |
|  | [1.16 - 1.25] | [1.14 - 1.21] | [0.96 - 1.45] | [1.22 - 1.52] | [0.40 - 0.69] | [0.30 - 0.40] |
| **Gender** |  |  |  |  |  |  |
| Male (ref) |  |  |  |  |  |  |
| Female |  | 1.20*** |  | 1.49*** |  | 0.66*** |
|  |  | [1.15 - 1.25] |  | [1.38 - 1.60] |  | [0.60 - 0.72] |
| **Relationship status** |  |  |  |  |  |  |
| Partnered (ref) |  |  |  |  |  |  |
| Unpartnered | 0.97** | 0.97** | 1.20*** | 1.36*** | 1.17* | 1.25*** |
|  | [0.95 - 0.99] | [0.95 - 0.99] | [1.08 - 1.34] | [1.27 - 1.45] | [1.03 - 1.33] | [1.15 - 1.35] |
| **Highest education level completed** |  |  |  |  |  |  |
| Year 12 and below (ref) |  |  |  |  |  |  |
| Professional qualifications | 0.99 | 1.00 | 1.09 | 1.14** | 1.32** | 1.20*** |
|  | [0.95 - 1.03] | [0.97 - 1.03] | [0.90 - 1.33] | [1.05 - 1.23] | [1.08 - 1.61] | [1.09 - 1.32] |
| University qualifications | 1.07* | 0.97 | 1.00 | 1.20*** | 3.64*** | 1.25*** |
|  | [1.01 - 1.12] | [0.94 - 1.01] | [0.81 - 1.24] | [1.09 - 1.32] | [2.77 - 4.79] | [1.10 - 1.41] |
| **Indigenous status** |  |  |  |  |  |  |
| Not of Indigenous origin (ref) |  |  |  |  |  |  |
| Indigenous origin |  | 1.26*** |  | 1.02 |  | 1.36* |
|  |  | [1.12 - 1.43] |  | [0.82 - 1.26] |  | [1.06 - 1.73] |
| **Region of residence** |  |  |  |  |  |  |
| Major city (ref) |  |  |  |  |  |  |
| Regional city | 0.96** | 0.98 | 1.02 | 0.93 | 1.02 | 1.02 |
|  | [0.93 - 0.99] | [0.96 - 1.01] | [0.87 - 1.20] | [0.87 - 1.00] | [0.84 - 1.22] | [0.93 - 1.11] |
| Remote area | 0.96 | 0.95 | 0.90 | 0.75* | 0.90 | 1.01 |
|  | [0.87 - 1.05] | [0.88 - 1.03] | [0.55 - 1.47] | [0.57 - 0.98] | [0.51 - 1.59] | [0.73 - 1.40] |
| **Household yearly disposable income** |  |  |  |  |  |  |
| Quintile 1 (Poorest) | 0.74*** | 0.74*** | 0.99 | 1.12* | 1.58*** | 2.36*** |
|  | [0.72 - 0.76] | [0.73 - 0.76] | [0.87 - 1.13] | [1.02 - 1.23] | [1.32 - 1.88] | [2.07 - 2.69] |
| Quintile 2 (Poorer) | 0.81*** | 0.82*** | 0.92 | 0.99 | 1.41*** | 2.02*** |
|  | [0.79 - 0.83] | [0.80 - 0.84] | [0.81 - 1.04] | [0.91 - 1.09] | [1.20 - 1.67] | [1.78 - 2.29] |
| Quintile 3 (Middle) | 0.87*** | 0.89*** | 0.89* | 0.93 | 1.24** | 1.61*** |
|  | [0.86 - 0.89] | [0.87 - 0.90] | [0.80 - 1.00] | [0.86 - 1.02] | [1.05 - 1.45] | [1.42 - 1.83] |
| Quintile 4 (Richer) | 0.97** | 0.99 | 0.95 | 0.98 | 1.06 | 1.23** |
|  | [0.96 - 0.99] | [0.97 - 1.00] | [0.86 - 1.05] | [0.90 - 1.06] | [0.91 - 1.24] | [1.08 - 1.40] |
| Quintile 5 (Richest) (ref) |  |  |  |  |  |  |
| **Health-related characteristics** |  |  |  |  |  |  |
| **BMI** |  |  |  |  |  |  |
| Underweight | 1.21*** | 1.15*** | 1.30 | 1.18 | 0.61** | 0.87 |
|  | [1.14 - 1.28] | [1.09 - 1.22] | [0.99 - 1.71] | [0.97 - 1.43] | [0.43 - 0.86] | [0.68 - 1.10] |
| Healthy weight (ref) |  |  |  |  |  |  |
| Overweight | 0.91*** | 0.94*** | 1.01 | 0.97 | 1.02 | 1.04 |
|  | [0.89 - 0.92] | [0.92 - 0.95] | [0.91 - 1.12] | [0.91 - 1.04] | [0.90 - 1.16] | [0.96 - 1.14] |
| Obese | 0.92*** | 0.99 | 1.10 | 1.00 | 1.05 | 1.19** |
|  | [0.89 - 0.94] | [0.96 - 1.01] | [0.94 - 1.28] | [0.93 - 1.09] | [0.87 - 1.26] | [1.07 - 1.32] |
| **Disability** |  |  |  |  |  |  |
| No (ref) |  |  |  |  |  |  |
| Yes | 1.33*** | 1.36*** | 1.32*** | 1.84*** | 1.03 | 1.10* |
|  | [1.31 - 1.35] | [1.34 - 1.38] | [1.20 - 1.44] | [1.72 - 1.97] | [0.91 - 1.16] | [1.01 - 1.21] |
| **Health-related behaviours** |  |  |  |  |  |  |
| **Smoking status** |  |  |  |  |  |  |
| Non-smoker (ref) |  |  |  |  |  |  |
| Current smoker | 0.89*** | 0.93*** | 1.32*** | 1.24*** | 1.07 | 1.52*** |
|  | [0.86 - 0.91] | [0.91 - 0.95] | [1.16 - 1.51] | [1.15 - 1.34] | [0.92 - 1.25] | [1.38 - 1.66] |
| **Alcohol consumption** |  |  |  |  |  |  |
| Non-drinker (ref) |  |  |  |  |  |  |
| Current drinker | 0.83*** | 0.84*** | 1.07 | 1.18*** | 1.16 | 1.02 |
|  | [0.81 - 0.85] | [0.83 - 0.86] | [0.93 - 1.22] | [1.08 - 1.28] | [0.98 - 1.37] | [0.92 - 1.14] |
| **Physical Activity** |  |  |  |  |  |  |
| Less than the recommended level (ref) |  |  |  |  |  |  |
| Recommended level | 0.94*** | 0.94*** | 0.87*** | 0.82*** | 1.10* | 1.13** |
|  | [0.92 - 0.95] | [0.92 - 0.95] | [0.80 - 0.94] | [0.77 - 0.87] | [1.00 - 1.21] | [1.05 - 1.21] |
| **Job-related characteristics** |  |  |  |  |  |  |
| **Firm size** |  |  |  |  |  |  |
| Small (1-19 employees) (ref) |  |  |  |  |  |  |
| Medium (20-99 employees) | 1.28*** | 1.34*** | 0.96 | 0.89*** | 0.81*** | 0.82*** |
|  | [1.26 - 1.30] | [1.32 - 1.37] | [0.88 - 1.06] | [0.83 - 0.95] | [0.73 - 0.90] | [0.75 - 0.89] |
| Large (≥100 employees) | 1.36*** | 1.44*** | 0.93 | 0.91* | 0.75*** | 0.77*** |
|  | [1.34 - 1.39] | [1.42 - 1.47] | [0.84 - 1.03] | [0.85 - 0.98] | [0.66 - 0.85] | [0.70 - 0.84] |
| **Employment contract** |  |  |  |  |  |  |
| Permanent (ref) |  |  |  |  |  |  |
| Fixed-term | 0.95*** | 0.95*** | 0.87* | 0.89* | 1.19* | 1.26*** |
|  | [0.93 - 0.97] | [0.93 - 0.97] | [0.78 - 0.98] | [0.80 - 0.98] | [1.02 - 1.38] | [1.11 - 1.43] |
| Casual | 0.54*** | 0.54*** | 0.88 | 0.88 | 1.31** | 1.38*** |
|  | [0.51 - 0.57] | [0.51 - 0.57] | [0.73 - 1.05] | [0.77 - 1.02] | [1.08 - 1.60] | [1.17 - 1.62] |
| **Occupation** |  |  |  |  |  |  |
| Professional (ref) |  |  |  |  |  |  |
| Manager | 0.92*** | 0.90*** | 0.92 | 0.93 | 0.72** | 0.72*** |
|  | [0.90 - 0.95] | [0.88 - 0.93] | [0.80 - 1.06] | [0.84 - 1.03] | [0.58 - 0.91] | [0.60 - 0.85] |
| Technician and trade workers | 1.14*** | 1.12*** | 0.88 | 0.75*** | 1.04 | 1.94*** |
|  | [1.10 - 1.17] | [1.09 - 1.16] | [0.74 - 1.05] | [0.67 - 0.84] | [0.84 - 1.30] | [1.67 - 2.24] |
| Community and personal service workers | 0.93*** | 0.95** | 0.83* | 0.81*** | 1.38** | 2.25*** |
|  | [0.90 - 0.97] | [0.92 - 0.98] | [0.71 - 0.98] | [0.73 - 0.91] | [1.12 - 1.69] | [1.95 - 2.60] |
| Clerical and administrative workers | 0.98 | 1.00 | 0.89 | 0.79*** | 1.08 | 1.32*** |
|  | [0.96 - 1.01] | [0.97 - 1.02] | [0.77 - 1.04] | [0.71 - 0.88] | [0.87 - 1.34] | [1.13 - 1.53] |
| Sales workers | 0.87*** | 0.87*** | 0.83* | 0.83** | 1.14 | 1.70*** |
|  | [0.84 - 0.90] | [0.84 - 0.90] | [0.70 - 0.98] | [0.73 - 0.93] | [0.91 - 1.42] | [1.45 - 1.99] |
| Machinery operators and drivers | 0.87*** | 0.90*** | 0.88 | 0.71*** | 1.15 | 2.33*** |
|  | [0.83 - 0.91] | [0.86 - 0.93] | [0.70 - 1.10] | [0.61 - 0.83] | [0.89 - 1.48] | [1.96 - 2.78] |
| Labourers | 0.83*** | 0.83*** | 0.89 | 0.71*** | 1.26* | 2.68*** |
|  | [0.80 - 0.87] | [0.80 - 0.86] | [0.74 - 1.08] | [0.62 - 0.81] | [1.01 - 1.57] | [2.30 - 3.13] |
| **Supervisory responsibilities** |  |  |  |  |  |  |
| Yes (ref) |  |  |  |  |  |  |
| No | 0.97*** | 0.97*** | 1.01 | 1.04 | 1.21*** | 1.21*** |
|  | [0.96 - 0.98] | [0.95 - 0.98] | [0.94 - 1.09] | [0.98 - 1.11] | [1.10 - 1.32] | [1.12 - 1.30] |
| **Paid holiday leave** |  |  |  |  |  |  |
| Yes (ref) |  |  |  |  |  |  |
| No | 0.74*** | 0.78*** | 0.93 | 1.09 | 1.10 | 1.09 |
|  | [0.68 - 0.80] | [0.72 - 0.84] | [0.65 - 1.34] | [0.82 - 1.46] | [0.73 - 1.67] | [0.78 - 1.52] |
| **Paid sick leave** |  |  |  |  |  |  |
| Yes (ref) |  |  |  |  |  |  |
| No | 0.36*** | 0.29*** | 1.26 | 1.11 | 1.21 | 1.27 |
|  | [0.33 - 0.39] | [0.26 - 0.31] | [0.88 - 1.82] | [0.83 - 1.48] | [0.80 - 1.82] | [0.91 - 1.78] |
| **Union membership** |  |  |  |  |  |  |
| Yes (ref) |  |  |  |  |  |  |
| No | 0.90*** | 0.86*** | 1.01 | 0.98 | 1.00 | 0.98 |
|  | [0.88 - 0.91] | [0.85 - 0.88] | [0.91 - 1.13] | [0.91 - 1.05] | [0.87 - 1.14] | [0.89 - 1.08] |
| **Overall job satisfaction** | 0.93*** | 0.94*** | 0.89*** | 0.88*** | 0.91*** | 0.88*** |
|  | [0.93 - 0.94] | [0.93 - 0.94] | [0.87 - 0.90] | [0.86 - 0.89] | [0.88 - 0.93] | [0.86 - 0.90] |
| Observations | n_observations_ = 52,314 | n_observations_ = 70,973 | n_observations_ = 31,285 | n_observations_ = 70,973 | n_observations_ = 17,143 | n_observations_ = 70,973 |
|  | n_individuals_ = 10,376 | n_individuals_ = 18,729 | n_individuals_ = 6,067 | n_individuals_ = 18,729 | n_individuals_ = 3,416 | n_individuals_ = 18,729 |
| Individual fixed effects | Yes |  | Yes |  | Yes |  |
| Wave fixed effects | Yes |  | Yes |  | Yes |  |

Notes: 1. 95% confidence intervals are reported in parentheses. 2. * indicates significance at the 5% level, **indicates significance at the 1% level, *** indicates significance at the 0.1% level. 3. Abbreviations: FE = Fixed-effects; RE = Random-effects; aOR = Adjusted Odds Ratio; aIRR = Adjusted Incidence Rate Ratio; Ref = reference category. 4. values are rounded off to two decimal places.

Table A3: The effects of psychological distress on the number of days worked despite feeling either physically or mentally unwell (an alternative measure of presenteeism), Complete regression results

| **Variables** | **Presenteeism** | |
| --- | --- | --- |
|  | **Model 1: Poisson regression** | **Model 4: Negative binomial regression** |
|  | **aIRR (95% CI)** | **aIRR (95% CI)** |
| **Psychological Distress** |  |  |
| Low (ref) |  |  |
| Moderate | 1.85*** | 1.86*** |
|  | [1.77 - 1.95] | [1.62 - 2.13] |
| High | 3.69*** | 4.02*** |
|  | [3.53 - 3.86] | [3.48 - 4.65] |
| **Socio-demographic characteristics** |  |  |
| **Age** |  |  |
| 17-24 years (ref) |  |  |
| 25-54 years | 1.04 | 1.13 |
|  | [0.99 - 1.10] | [0.94 - 1.37] |
| 55-67 years | 0.97 | 1.06 |
|  | [0.91 - 1.04] | [0.85 - 1.32] |
| **Relationship status** |  |  |
| Partnered (ref) |  |  |
| Unpartnered | 1.12*** | 1.14* |
|  | [1.08 - 1.16] | [1.00 - 1.29] |
| **Highest education level completed** |  |  |
| Year 12 and below (ref) |  |  |
| Professional qualifications | 1.06* | 1.06 |
|  | [1.01 - 1.10] | [0.91 - 1.23] |
| University qualifications | 0.86*** | 0.79* |
|  | [0.82 - 0.91] | [0.66 - 0.95] |
| **Region of residence** |  |  |
| Major city (ref) |  |  |
| Regional city | 1.07*** | 1.05 |
|  | [1.03 - 1.11] | [0.93 - 1.19] |
| Remote area | 1.96*** | 2.05** |
|  | [1.74 - 2.22] | [1.25 - 3.39] |
| **Household yearly disposable income** |  |  |
| Quintile 1 (Poorest) | 1.13*** | 1.09 |
|  | [1.07 - 1.20] | [0.90 - 1.33] |
| Quintile 2 (Poorer) | 1.12*** | 1.06 |
|  | [1.05 - 1.18] | [0.88 - 1.28] |
| Quintile 3 (Middle) | 1.13*** | 1.01 |
|  | [1.07 - 1.20] | [0.84 - 1.22] |
| Quintile 4 (Richer) | 1.16*** | 1.08 |
|  | [1.10 - 1.23] | [0.91 - 1.29] |
| Quintile 5 (Richest) (ref) |  |  |
| **Health-related characteristics** |  |  |
| **BMI** |  |  |
| Underweight | 0.99 | 1.06 |
|  | [0.86 - 1.15] | [0.66 - 1.71] |
| Healthy weight (ref) |  |  |
| Overweight | 0.98 | 0.99 |
|  | [0.94 - 1.02] | [0.87 - 1.14] |
| Obese | 1.14*** | 1.16* |
|  | [1.09 - 1.19] | [1.00 - 1.35] |
| **Disability** |  |  |
| No (ref) |  |  |
| Yes | 1.93*** | 2.06*** |
|  | [1.86 - 2.00] | [1.79 - 2.38] |
| **Health-related behaviours** |  |  |
| **Smoking status** |  |  |
| Non-smoker (ref) |  |  |
| Current smoker | 1.23*** | 1.30** |
|  | [1.18 - 1.28] | [1.11 - 1.53] |
| **Alcohol consumption** |  |  |
| Non-drinker (ref) |  |  |
| Current drinker | 1.02 | 1.12 |
|  | [0.97 - 1.08] | [0.95 - 1.33] |
| **Physical Activity** |  |  |
| Less than the recommended level (ref) |  |  |
| Recommended level | 0.99 | 0.98 |
|  | [0.96 - 1.03] | [0.87 - 1.10] |
| **Job-related characteristics** |  |  |
| **Firm size** |  |  |
| Small (1-19 employees) (ref) |  |  |
| Medium (20-99 employees) | 0.90*** | 0.90 |
|  | [0.87 - 0.94] | [0.78 - 1.03] |
| Large (≥100 employees) | 0.92*** | 0.90 |
|  | [0.88 - 0.96] | [0.78 - 1.03] |
| **Employment contract** |  |  |
| Permanent (ref) |  |  |
| Fixed-term | 0.87*** | 1.03 |
|  | [0.81 - 0.93] | [0.82 - 1.28] |
| Casual | 0.80*** | 0.80 |
|  | [0.71 - 0.90] | [0.57 - 1.14] |
| **Occupation** |  |  |
| Professional (ref) |  |  |
| Manager | 1.19*** | 1.16 |
|  | [1.13 - 1.26] | [0.96 - 1.42] |
| Technicians and trade workers | 0.84*** | 0.84 |
|  | [0.78 - 0.90] | [0.67 - 1.05] |
| Community and personal service workers | 0.86*** | 0.77* |
|  | [0.81 - 0.92] | [0.62 - 0.95] |
| Clerical and administrative workers | 0.90*** | 0.84 |
|  | [0.84 - 0.96] | [0.68 - 1.03] |
| Sales workers | 0.80*** | 0.78 |
|  | [0.74 - 0.87] | [0.60 - 1.01] |
| Machinery operators and drivers | 0.90** | 0.88 |
|  | [0.83 - 0.97] | [0.67 - 1.16] |
| Labourers | 0.93 | 0.77 |
|  | [0.86 - 1.00] | [0.59 - 1.02] |
| **Supervisory responsibilities** |  |  |
| Yes (ref) |  |  |
| No | 0.81*** | 0.79*** |
|  | [0.78 - 0.84] | [0.70 - 0.90] |
| **Paid holiday leave** |  |  |
| Yes (ref) |  |  |
| No | 0.71** | 0.92 |
|  | [0.57 - 0.89] | [0.45 - 1.87] |
| **Paid sick leave** |  |  |
| Yes (ref) |  |  |
| No | 1.36** | 1.09 |
|  | [1.09 - 1.70] | [0.54 - 2.20] |
| **Union membership** |  |  |
| Yes (ref) |  |  |
| No | 0.96 | 0.89 |
|  | [0.92 - 1.00] | [0.77 - 1.03] |
| **Overall job satisfaction** | 0.88*** | 0.88*** |
|  | [0.87 - 0.89] | [0.85 - 0.91] |
| Observations | n_observations_ = 31,285 | n_observations_ = 70,973 |
|  | n_individuals_ = 6,067 | n_individuals_ = 18,729 |

Notes: 1. 95% confidence intervals are reported in parentheses. 2. * indicates significance at the 5% level, **indicates significance at the 1% level, *** indicates significance at the 0.1% level. 3. Abbreviations: aIRR = Adjusted Incidence Rate Ratio; Ref = reference category. 4. values are rounded off to two decimal places.

Table A4: Additional sick leave days and number of days worked despite feeling unwell of an individual with moderate to high psychological distress compared to their counterparts without or low psychological distress by three measures

| **Types of productivity loss** | **Psychological distress** | |
| --- | --- | --- |
|  | **Moderate** | **High** |
| **Additional sick leave days in the past 12 months** | | |
| Mean comparison, unadjusted | 0.43 days | 0.58 days |
| Assuming zero random effects, adjusted | 0.22 days | 0.36 days |
| **The additional number of days worked despite feeling unwell in the past 12 months** | | |
| Mean comparison, unadjusted | 4.68 days | 16.64 days |
| Assuming zero random effects, adjusted | 4.23 days | 13.26 days |

Source: Authors’ own calculations

Notes: 1. We assumed that a working adult has accomplished 50% of work/other activities as carefully as usual in a day when working despite being unwell. 2. We multiplied the additional number of days worked despite feeling unwell by 13 to get the value for the previous 12 months.

Table A5: Weekly and daily gross wages and salary according to gender in Australia

| **Gross wages and salary by gender** | **Sources** | |
| --- | --- | --- |
|  | **HILDA sample** | **ABS statistics** |
| **Overall** |  |  |
| Weekly | A$ 1,113.64 | A$ 1,378.60 |
| Daily | A$ 222.73 | A$ 275.72 |
| **Male** |  |  |
| Weekly | A$ 1,300.13 | A$ 1,621.20 |
| Daily | A$ 260.03 | A$ 324.24 |
| **Female** |  |  |
| Weekly | A$ 924.12 | A$ 1,146.10 |
| Daily | A$ 184.82 | A$ 229.22 |

Notes: 1. Authors have calculated weekly gross wages and salary using the HILDA sample. 2. For comparison purposes, the authors have accessed Australian Bureau of Statistics (ABS) data on gross wages and salary from the following web link: https://www.abs.gov.au/statistics/labour/earnings-and-working-conditions/average-weekly-earnings-australia/nov-2022 3. Daily gross wages and salary were calculated by dividing weekly gross wages and salary by 5. 4. Abbreviation: A$ = Australian Dollar.

Table A6: Sensitivity analyses of costs of presenteeism attributed to psychological distress according to gender

| **Types of productivity costs** | **Unit wages estimated from the HILDA sample** | | | **Unit wages according to ABS** | | |
| --- | --- | --- | --- | --- | --- | --- |
|  | **Male** | **Female** | **Overall** | **Male** | **Female** | **Overall** |
| **Attributable costs of yearly presenteeism (assuming that a working individual accomplished 40% of work in a day when working despite being unwell) due to psychological distress** | | | | | | |
| **Mean comparison, unadjusted** | | | | | | |
| Moderate psychological distress | 972.51 | 691.23 | 833.01 | 1,212.66 | 857.28 | 1,031.19 |
| High psychological distress | 3,461.00 | 2,459.95 | 2,964.54 | 4,315.63 | 3,050.92 | 3,669.83 |
| **Zero random effects, adjusted** | | | | | | |
| Moderate psychological distress | 878.90 | 624.69 | 752.83 | 1,095.93 | 774.76 | 931.93 |
| High psychological distress | 2,758.92 | 1,960.94 | 2,363.17 | 3,440.19 | 2,432.02 | 2,925.39 |
| **Attributable costs of yearly presenteeism (assuming that a working individual accomplished 60% of work in a day when working despite being unwell) due to psychological distress** | | | | | | |
| **Mean comparison, unadjusted** | | | | | | |
| Moderate psychological distress | 1,461.37 | 1,038.69 | 1,251.74 | 1,822.23 | 1,288.22 | 1,549.55 |
| High psychological distress | 5,192.80 | 3,690.86 | 4,447.92 | 6,475.07 | 4,577.52 | 5,506.13 |
| **Zero random effects, adjusted** | | | | | | |
| Moderate psychological distress | 1,318.35 | 937.04 | 1,129.24 | 1,643.90 | 1,162.15 | 1,397.90 |
| High psychological distress | 4,137.08 | 2,940.49 | 3,543.63 | 5,158.66 | 3,646.89 | 4,386.71 |

Source: Authors’ own calculations

Notes: 1. Values for each cell were obtained by multiplying respective daily wages and salary with additional absent days. Please refer to Appendix Tables 3 and 4 for gross wages and salary, and additional absent days, respectively. 3. Abbreviation: ABS = Australian Bureau of Statistics. 4. All values are reported in Australian dollars and were rounded into two decimal points.
